# The circadian clock gene BMAL1 modulates autoimmunity features in lupus

**DOI:** 10.3389/fimmu.2024.1465185

**Published:** 2024-11-27

**Authors:** Shuichiro Nakabo, Donavon Sandoval-Heglund, Norio Hanata, Stephen Brooks, Victoria Hoffmann, Mingzeng Zhang, William G. Ambler, Zerai Manna, Elaine Poncio, Sarfaraz Hasni, Shamima Islam, Stefania Dell’Orso, Mariana J. Kaplan

**Affiliations:** 1 Systemic Autoimmunity Branch, National Institute of Arthritis and Musculoskeletal and Skin Diseases (NIAMS), National Institutes of Health (NIH), Bethesda, MD, United States; 2 Biodata Mining and Discovery Section, NIAMS, NIH, Bethesda, MD, United States; 3 Division of Veterinary Resources, NIH, Bethesda, MD, United States; 4 Lupus Clinical Trials Unit, NIAMS, NIH, Bethesda, MD, United States; 5 Genomic Technology Section, Office of Science and Technology, NIAMS, NIH, Bethesda, MD, United States

**Keywords:** systemic lupus erythematosus, neutrophils, autoantibody, clock gene, Bmal1, April

## Abstract

**Objectives:**

An important pathogenic role for neutrophils in systemic lupus erythematosus (SLE) has been proposed. Neutrophils that lack brain and muscle aryl hydrocarbon receptor nuclear translocator-like 1 (*Bmal1*), one of the clock genes, are defective in aging and proinflammatory responses. We assessed the role of *Bmal1* in clinical and immunologic manifestations of murine lupus and in human SLE neutrophils.

**Methods:**

Myeloid-conditional *Bmal1* knockout mice (*Bmal1^Mye−/−^*) and wild type (WT) were treated with epicutaneous TLR7/8 agonist (imiquimod; IMQ) for 6 weeks to induce a lupus phenotype. Upon euthanasia, immune responses, autoantibodies and renal manifestations were evaluated. NET formation and gene expression of bone marrow (BM)-derived murine neutrophils were evaluated. *BMAL1* expression was quantified in SLE neutrophils and compared with clinical disease.

**Results:**

IMQ-treated *Bmal1^Mye−/−^* and WT displayed comparable systemic inflammation. While renal function did not differ, serum anti-dsDNA levels and renal immune complex deposition were significantly increased in *Bmal1^Mye−/−^*. While no differences were observed in NET formation, expression levels of *April* in BM neutrophils were significantly higher in *Bmal1^Mye−/−^*. Bulk RNA-sequence data showed that BM neutrophils in IMQ-treated *Bmal1^Mye−/−^* were relatively immature when compared with IMQ-treated WT. BM showed an enhanced April protein expression in *Bmal1^Mye−/−^* mice. *BMAL1* levels in human SLE peripheral blood neutrophils correlated positively with serum C3 and negatively with serum anti-dsDNA levels.

**Conclusion:**

*Bmal1* is associated with lower disease activity in SLE. These results indicate that perturbation in the circadian rhythm of neutrophils can have pathogenic consequences in SLE.

## 1 Introduction

Systemic lupus erythematosus (SLE) is an autoimmune syndrome of unclear etiology that affects multiple organs and systems, including kidney, the vasculature, and the skin. Recent evidence supports a role for neutrophil dysregulation in lupus pathogenesis. Part of this evidence comes from the observation of perturbed neutrophil extracellular trap (NET) formation and clearance in SLE. NET formation is characterized by the extrusion of neutrophil nucleic acids and antibacterial components to the extracellular space and represents a process of programmed cell death that can promote autoantigen externalization and modification as well as tissue damage (1). Neutrophils also synthesize various cytokines that have potential pathogenic impact in SLE, including type I interferons (IFNs), B-cell activating factor (BAFF), and a proliferation-inducing ligand (APRIL) (2).

Neutrophils differentiate in the bone marrow and egress into the circulation following a circadian rhythm pattern. Aging of peripheral neutrophils is influenced by one of the clock genes, brain and muscle aryl hydrocarbon receptor nuclear translocator-like 1 (*Bmal1*) (3). Neutrophil aging is induced by the C–X–C chemokine receptor (Cxcr) 2, whereas Bmal1 positively regulates the Cxcr2 pathway by driving C–X–C chemokine ligand (Cxcl) 2 expression. Therefore, neutrophils that lack Bmal1 display defects in aging. Because young neutrophils have higher migratory capabilities into inflammatory sites and mice that specifically lack Bmal1 in neutrophils are more resistant to infection (3), we hypothesized that lack of Bmal1 could result in worsening of lupus features by modulating neutrophil aging and enhance inflammatory responses.

While the roles of clock genes and the effects of disruption of circadian rhythms in SLE are largely unknown, immune responses are known to be modulated by circadian rhythms and many diseases, including inflammatory diseases, show circadian oscillation in their severity (4). In mice genetically prone to develop SLE-like manifestations, sleep deprivation induced earlier induction of autoantibodies (5), whereas several studies have suggested that shorter sleep duration is associated with SLE diagnosis (6, 7). While these findings suggest that disruption of circadian rhythms may be involved in SLE pathogenesis, a systematic assessment of the role of clock genes in SLE pathogenesis remains to be reported.

Here, we tested the hypothesis that disruptions in circadian rhythm through clock gene dysregulation could modulate lupus autoimmunity. We used an induced model of murine lupus triggered by epicutaneous administration of the TLR7/8 agonist imiquimod (IMQ) (8) and investigated lupus phenotype in wild type (WT) and Bmal1-myeloid conditional KO mice (*Bmal1^Mye−/−^*). We observed that lack of Bmal1 in the myeloid compartment associated with positive regulation of autoantibody production and immune complex deposition, in association with increases in *April* expression in bone marrow neutrophils. We also found an association between *BMAL1* expression in circulating neutrophils and clinical/serological parameters of disease activity in human SLE, supporting a role for circadian rhythm disturbances in autoimmunity development and perpetuation.

## 2 Materials and methods

### 2.1 Mice

Wild-type (WT; C57BL/6J) mice were purchased from the Jackson Laboratory. Breeding pairs of Bmal1-myeloid conditional knockout (*Bmal1^Mye−/−^*; Bmal1FloxP/FloxP;LysMCre) mice were created by breeding B6.129S4(Cg)-Bmal1^tm1Weit^/J (JAX007668) with B6.129P2-Lyz2^tm1(cre)Ifo^/J (JAX004781) and kept at the National Institute of Arthritis and Musculoskeletal and Skin Diseases (NIAMS) animal facility. In total, 74 WT and 70 *Bmal1^Mye−/−^* mice (all female) were used for the experiments. Each dot in the figures reporting animal experiments represents an individual. All mice were kept under specific pathogen-free conditions, and all procedures were performed in accordance with NIH guidelines and approved by the NIAMS Animal Care and Use Committee (ACUC) (protocol numbers are A019-05-03 and A022-08-04). All animals were euthanized in a CO_2_ chamber, followed by cervical dislocation once they stopped breathing. No anesthesia was used for the procedures.

### 2.2 TLR7/8-induced lupus model

Imiquimod (IMQ) cream (0.125 g of 5%; Fougera or Perrigo) was applied on the dorsal side of both ears of 8–10-week-old female mice, three times per week for 6 weeks, as previously described (8). The baseline body weight of the mice was around 20 g. Treatment was done in the evening (around Zeitgeber time (ZT) 11). Blinding or randomization for treatment was not performed. Peripheral blood, spleen, kidney, and ear skin samples were harvested at euthanasia. Mouse euthanasia was done in the morning (ZT 1–4) for all experiments. Animals who died before completing a 6-week treatment period were excluded from the analysis except for survival analysis.

### 2.3 Histological evaluation

Mouse kidney, ear pinna, and sternum bone marrow were harvested and fixed with 10% formalin at euthanasia. Samples were stored in 10% formalin until slides were prepared. Samples were stained with hematoxylin and eosin (H&E) for ear pinna or H&E and periodic acid–Schiff stain (PAS) for kidneys. The severity of inflammation was semiquantitatively scored by a veterinary pathologist (VH) blinded to background information and sample identity. The number of infiltrating neutrophils was counted on five randomly selected high-power fields of H&E-stained slides. Scoring was performed as follows: for kidneys, glomeruli ware scores on PAS-stained slides. The total scores were generated by adding up the severity and distribution score for hypercellularity, mesangial matrix expansion, and inflammation. The definition of distribution was as follows: 0 = <1%, 1 = 1%–25%, 2 = 25%–75%, 3 = >75%. The definition of severity was as follows: 0 = no significant findings (within normal limits), 1 = mild (focal segmental), 2 = moderate (multifocal segmental), 3 = severe (global) (9). The detailed explanation of each score was provided in **Supplementary Table 1**.

For ear pinna, the severity and distribution in the epidermis and dermis were scored. For epidermis, the score is as follows: 0 = normal, 1 = mild (hyperplasia two to three layers thick), 2 = moderate (acanthosis with an occasional increase in thickness of the granular layer), 3 = severe (dyskeratosis, diffuse increase in granular layer, hyperkeratosis rete peg formation). For dermis, the score is as follows: 0 = normal, 1 = mild (slight increase in cellularity; perivascular cuffs), 2 = moderate (definite increase in cellularity; fibrotic foci, folliculitis), 3 = severe (pustules; extensive fibrosis, vacuoles/clefting associated with basement membrane).

For bone marrow immunohistochemistry (IHC), slides were treated with H_2_O_2_ to block endogenous peroxidases and incubated with Polyclonal Rabbit anti−Human TNFSF13/APRIL Antibody (aa151−200) (LS Bio) at 10 μg/mL. April was visualized with anti-rabbit-HRP secondary antibody and 3,3′-diaminobenzidine (DAB). Finally, slides were counterstained with hematoxylin, dehydrated, and cleared in graded alcohols and xylene, respectively. Images were obtained with a NanoZoomer-XR digital slide scanner (Hamamatsu). Whole bone marrow images were exported as TIFF files, and April-positive area was quantified by ImageJ ver. 1.54i 03.

### 2.4 RNA extraction, cDNA synthesis, and quantitative PCR

Tissue specimens were put in RNAlater (Thermo Fisher Scientific) at euthanasia of mice and stored at −80°C until they were processed. They were frozen in liquid nitrogen, ground into a fine powder by pestle and mortar, and lysed by TRIzol reagent (Thermo Fisher Scientific). Cell samples were lysed by TRIzol reagent immediately after isolation and stored at −80°C. RNA was purified by using Direct-zol RNA kits (Zymo Research) following the manufacturer’s instructions and quantified by NanoDrop Microvolume Spectrophotometer (Thermo Fisher Scientific). The quality of RNA samples was evaluated by the ratio of absorbance at 260 to 280. cDNA was synthesized by iScript reverse transcription supermix (Bio-Rad). Quantitative PCR (qPCR) was performed on a CFX96 real-time PCR detection system (Bio-Rad) with TaqMan gene expression assays (Thermo Fisher Scientific). Expression of each gene was normalized to Gapdh, and fold change was calculated using the ΔΔCt method. *Cxcl1* and *Cxcl2* expression in ear skin were evaluated as they are known chemoattractants of neutrophils. *Irf7* and *Isg15* in kidney were evaluated as IFN-regulated genes and based on our previous reports using the same murine lupus model (10, 11). Bone marrow neutrophil *Baff*, *April*, and *Ifna4* were evaluated based on previous reports (2).

### 2.5 Isolation of bone marrow neutrophils and monocytes of mice

Mouse bone marrow cells were harvested from bilateral femurs and tibias. Neutrophils and monocytes were isolated from bone marrow cells using MACS-negative selection with a mouse neutrophil isolation kit and mouse monocyte isolation kit (BM) (Miltenyi Biotec), respectively. Purity of CD11b^+^Ly6G^+^ neutrophils was assessed using flow cytometry and was found to be higher than 95%.

### 2.6 Quantification of NETs

The NET-forming capacity of bone marrow neutrophils was assessed by the IncuCyte S3 Live-Cell Analysis System (Sartorius), as previously described (12, 13). In brief, nuclei of neutrophils were stained with NUCLEAR-ID Red DNA stain (Enzo), and 15,000/well of neutrophils were plated onto a 96-well tissue culture-treated plate (Corning). Cells were then incubated in serum-free RPMI-1640 media (Thermo Fisher Scientific) with 1:25,000 diluted SYTOX Green (Thermo Fisher Scientific) for 16 hours at 37֯°C with 5% CO_2_. For stimulation, 25 nM phorbol 12-myristate 13-acetate (PMA; MilliporeSigma) or 2.5 µM calcium ionophore A23187 (MilliporeSigma) was used. SYTOX Green-positive cells are defined as NET-forming neutrophils and percentage was assessed every 20 min by time-lapse imaging. In order to minimize the influence from inter-assay variability, equal numbers of samples from each group were included in the same plate and evaluated at the same time.

### 2.7 Immunofluorescent staining of NETs

Immunofluorescent staining of NETs was performed as we previously reported (14). Bone marrow neutrophils were seeded on poly-L-lysine–coated coverslip and incubated for 5 h in RPMI without serum at 37°C in a CO_2_ chamber. They were then fixed with 4% paraformaldehyde overnight at 4°C. Subsequently, coverslips were washed by PBS twice and blocked by 0.2% gelatin for 1 h at room temperature, incubated with 1:100 diluted rabbit polyclonal anti-histone H3 (citrulline R2 + R8 + R17) antibody (Abcam), washed three times, and stained with 1:400 diluted Alexa Fluor 555-conjugated donkey anti-Rabbit IgG (H+L) Antibody (Thermo Fisher Scientific). After washing, cells were counterstained with 1:1,000 diluted Hoechst (Thermo Fisher Scientific) and washed twice, and then visualized using a Leica DMI4000 B inverted microscope (Leica Microsystems). Specificity of the primary antibody was confirmed by staining only with the secondary antibody.

### 2.8 Quantification of serum NET complexes

Serum citrullinated histone H3-DNA and neutrophil elastase–DNA complex were measured by sandwich ELISA as previously described (15). In short, high-binding 96-well ELISA plates (Corning) were coated by either 75 µL/well of 2.5 μg/mL rabbit anti-citrullinated histone H3 antibody (Abcam) or rabbit anti-neutrophil elastase antibody (Abcam). After overnight incubation at 4°C, the plates were blocked by 100 μL/well of 1% BSA-PBS at room temperature for 1 h. Subsequently, mouse serum samples (1:100 diluted by 1% BSA-PBS) were applied to each well and incubated overnight at 4°C. Next, plates were washed by 200 μL/well PBS containing 0.05% Tween 20 (PBS-T) three times; they were incubated with mouse anti-ds DNA monoclonal antibody (100 μL/well, 1:100 diluted by 1% BSA-PBS; EMD Millipore) for 1 h at room temperature. The plates were washed three times with 200 μL/well PBS-T, and HRP-conjugated anti-mouse antibody (100 μL/well, 1:10,000 diluted by 1% BSA-PBS; Bio-Rad) was added to each well. Finally, the plates were washed five times with 200 μL/well PBS-T, and ELISA was visualized with tetramethylbenzidine and stop solution.

### 2.9 Assessment of NETs in skin lesion by immunofluorescent staining

Mouse ear pinna was snap frozen in an OCT compound (Tissue-Tek). Frozen sections were blocked with 1% BSA-PBS for 1 h at room temperature. Sections were incubated overnight at 4°C with rabbit polyclonal anti-histone H3 (citrulline R2 + R8 + R17) antibody (Abcam), which was 1:500 diluted using 1% BSA-PBS. After washing five times, they were stained with 1:400 diluted Alexa Fluor 555-conjugated donkey anti-Rabbit IgG (H+L) Antibody (Thermo Fisher Scientific) for 1 h at room temperature. Slides were then counterstained with Hoechst (Thermo Fisher Scientific) for 10 min at room temperature, washed, mounted by Prolong Gold (Thermo Fisher Scientific), and visualized using a Leica DMI4000 B inverted microscope (Leica Microsystems).

### 2.10 Assessment of immune complex deposition in kidney by immunofluorescent staining

Mouse kidney was snap-frozen in an OCT compound (Tissue-Tek). Frozen sections were fixed with cold acetone for 10 min at −20°C, washed, and blocked by 5% BSA-PBS at room temperature for 1 h. Subsequently, they were stained with Alexa Fluor 594-conjugated goat anti-mouse IgG (1:100 diluted by PBS; Thermo Fisher Scientific), FITC-conjugated goat anti-mouse C3 antibody (1:100 diluted by PBS; Immunology Consultants Laboratory), and Hoechst (1:1,000 diluted by PBS; Thermo Fisher Scientific) at room temperature for 1.5 h. After washing, they were mounted with Prolong Gold (Thermo Fisher Scientific) and visualized using a Leica DMI4000 B inverted microscope (Leica Microsystems).

For quantification of immune complex deposition, three photos per sample were taken to include four glomeruli. Integrated density was calculated by ImageJ 2.3.0. Average integrated density of three photos from each sample was compared.

### 2.11 Quantification of mouse serum autoantibodies, total IgG, April, cell-free DNA, and DNase I activity

Serum autoantibodies, total IgG, April, cell-free DNA, and DNase I activity were quantified by commercially available ELISAs or plate assay kits. Mouse serum anti-dsDNA, anti-RNP, and anti-SS-A/Ro antibodies were assessed by Mouse anti-dsDNA IgG-specific ELISA Kit, Mouse Anti-nRNP Igs (total (A+G+M)) ELISA Kit, and Mouse Anti-SSA/Ro60 Ig’s (total) ELISA Kit, respectively (all from Alpha Diagnostic). Total serum IgG concentrations were determined by IgG (Total) Mouse Uncoated ELISA Kit (Thermo Fisher Scientific). Serum April levels were tested by Mouse TNFSF13/APRIL ELISA Kit (LS Bio). Cell-free DNA in mouse peripheral blood was analyzed with the Circulating DNA Quantification Kit (Abcam). Serum DNase I activity was tested with the DNase I assay kit (Abcam).

### 2.12 Assessment of urine albumin and creatinine

Mouse urine samples were collected at euthanasia and frozen at −80°C until used. Urine albumin and creatinine were quantified by Albuwell M and the Creatinine Companion (both from Ethos Biosciences). The severity of proteinuria was assessed by dividing urine albumin level by urine creatinine level.

### 2.13 Flow cytometry

Splenocytes were harvested at euthanasia and immediately incubated with TruStain FcX (BioLegend) for 15 min at 4°C. Subsequently, fluorochrome-conjugated antibodies were added, and cells were incubated for 30 min in 4°C. Splenocytes were then washed with PBS and stained with LIVE/Dead Fixable Aqua (Thermo Fisher Scientific) for 30 min in the dark at room temperature, in accordance with the manufacturer’s instructions. After washing, they were promptly analyzed on a BD FACSCelesta flow cytometer (Becton, Dickinson & Company). Data were analyzed using FlowJo 10.8.1 (Becton, Dickinson & Company). Gating strategies were established based on previous reports (11, 16). The number of cells was counted using CountBright Plus Absolute Counting Beads (Thermo Fisher Scientific). A detailed information of antibodies and gating strategy used are provided in a **Supplementary Excel File**.

### 2.14 RNA sequencing

#### 2.14.1 Sequencing

For transcriptome analysis, Illumina RNA libraries were prepared with the NEBNext Ultra II RNA library preparation kit for Illumina (NEB #E7490) according to the manufacturer’s instructions. Briefly, 500 ng of total RNA was enriched for poly (A)+ mRNA and retrotranscribed. cDNAs were then fragmented and adapters added to each end of the fragments. The obtained libraries were amplified and size-selected prior to NGS analysis. All libraries were diluted to 3 nM and sequenced on an Illumina NovaSeq 6000 or Illumina NextSeq 550 using the following read length: 50 bp for Read1, 8 bp for I7 Index, and 50 bp for Read2.

#### 2.14.2 Analysis

For each sample, Read 1 was mapped to mm10 using TopHat 2.1.1 and then resultant BAM files were imported into Partek Genomics Suites 7.0. RPKM values were calculated, log2 transformed with a 0.1 RPKM offset, and ANOVA comparisons calculated using Partek GS.

Pathway analysis was performed using differentially expressed genes with 2- or −2-fold changes, using Enrichr with BioPlanet 2019 (17, 18). The gene set to assess neutrophil maturation was based on a previous report (19). Library quality was determined by looking at mapping efficiency (BAM alignment count/FastQ read count) of >90%. FastQC was performed, and sequencing quality was found to be consistently optimal.

### 2.15 Immature neutrophil score

The immature neutrophil score was calculated as previously described (20, 21) with some modifications. In brief, RPKM values of seven genes were used for calculation (*Mpo*, *Elane*, *Bpi*, *Ctsg*, *Prtn3*, *Camp*, and *Defa4*). *AZU1* was removed because this gene is not present in mice. The mean value of each gene in untreated WT was subtracted from each value of each gene in each sample. Each value was then divided by the standard deviation of each calculated gene by values in untreated WT. The immature neutrophil score of each sample was calculated by the addition of these numbers. The qPCR version of immature neutrophil score was calculated by the addition of geometric mean of relative expression level of each gene (fold change based on the ΔΔCt method).

### 2.16 Analysis of plasma cell survival in culture supernatant of neutrophil

Bone marrow neutrophils were isolated from IMQ-treated *Bmal1^Mye−/−^* and WT using the method explained above (MACS-negative selection). The neutrophils were resuspended in RPMI with 10% fetal bovine serum (FBS) at 10 million neutrophils/mL and incubated for 16 h at 37°C with 5% CO_2_. Then, culture supernatant was collected and stored in −80°C until they were used.

IgD^−^ Lin (TER-119, F4/80, CD8a, CD4)^−^ CD138^+^ plasma cells were sorted out using FACSAria Fusion (Becton, Dickinson & Company) from spleen cells of untreated WT mice. The gating strategy was decided by referencing a previous report (22). After the sorting, plasma cells were resuspended in RPMI with 10% FBS at 1 × 10^5^ cells/mL and mixed with SYTOX Green and Incucyte Nuclight Rapid Red (Sartorius). Then, 100 µL of the cell suspension was mixed with the same volume of culture supernatant of neutrophils and plated onto a 96-well plate (Corning) with or without 50 μg/mL of anti-mouse April antibody (Apry-1-1, AdipoGen Life Sciences), which selectively inhibits the binding of April to its receptors BCMA and TACI (23, 24). Cell death was quantified using the same method as the quantification of NETs explained above (12, 25).

### 2.17 Human peripheral neutrophils and peripheral blood mononuclear cells, and clinical and peripheral blood parameters

Patients were recruited at the Clinical Center, NIH, Bethesda, MD, with written informed consent. The study is in compliance with the Helsinki Declaration and was approved by the institutional review board of NIAMS (NIH 94-AR-0066). SLE patients fulfilled the ACR/EULAR 2019 classification criteria for SLE. All blood samples were harvested in the morning (7–11 am). Samples were immediately processed. Neutrophils and peripheral blood mononuclear cells (PBMCs) were isolated by Ficoll-Paque (GE) density gradient and dextran (Alfa Aesar) sedimentation (25). The expression of each gene was normalized to *GAPDH*, and fold change was calculated using the ΔΔCt method. SLE disease activity index (SLEDAI), anti-dsDNA antibody, and complement C3 and C4 were quantified on the same day when each sample was taken. The positivity of anti-RNP, Sm, SS-A/Ro, and SS-B/La antibodies was based on the medical record review. Plasma APRIL levels were tested by Human APRIL/TNFSF13 DuoSet ELISA kit (R&D Systems).

### 2.18 Statistical analysis

Statistical analysis was done, and graphs were drawn by GraphPad Prism version 9.3.1. To compare two groups, unpaired t-test and Mann–Whitney test were used for parametric and non-parametric parameters, respectively. Correlation was evaluated with Pearson correlation or Spearman’s rank correlation coefficient. Log-rank test with Kaplan–Meier curve was used for mouse survival analysis. Repeated measures two-way ANOVA was used for plasma cell survival analysis. Adjustment for multiple comparison was not performed except for plasma cell survival assay. In any statistical test, p<0.05 was considered significant.

## 3 Results

### 3.1 *Bmal1^Mye−/−^* mice display increased serum anti-dsDNA antibody levels and renal immune complex deposition

**Figure 1:**
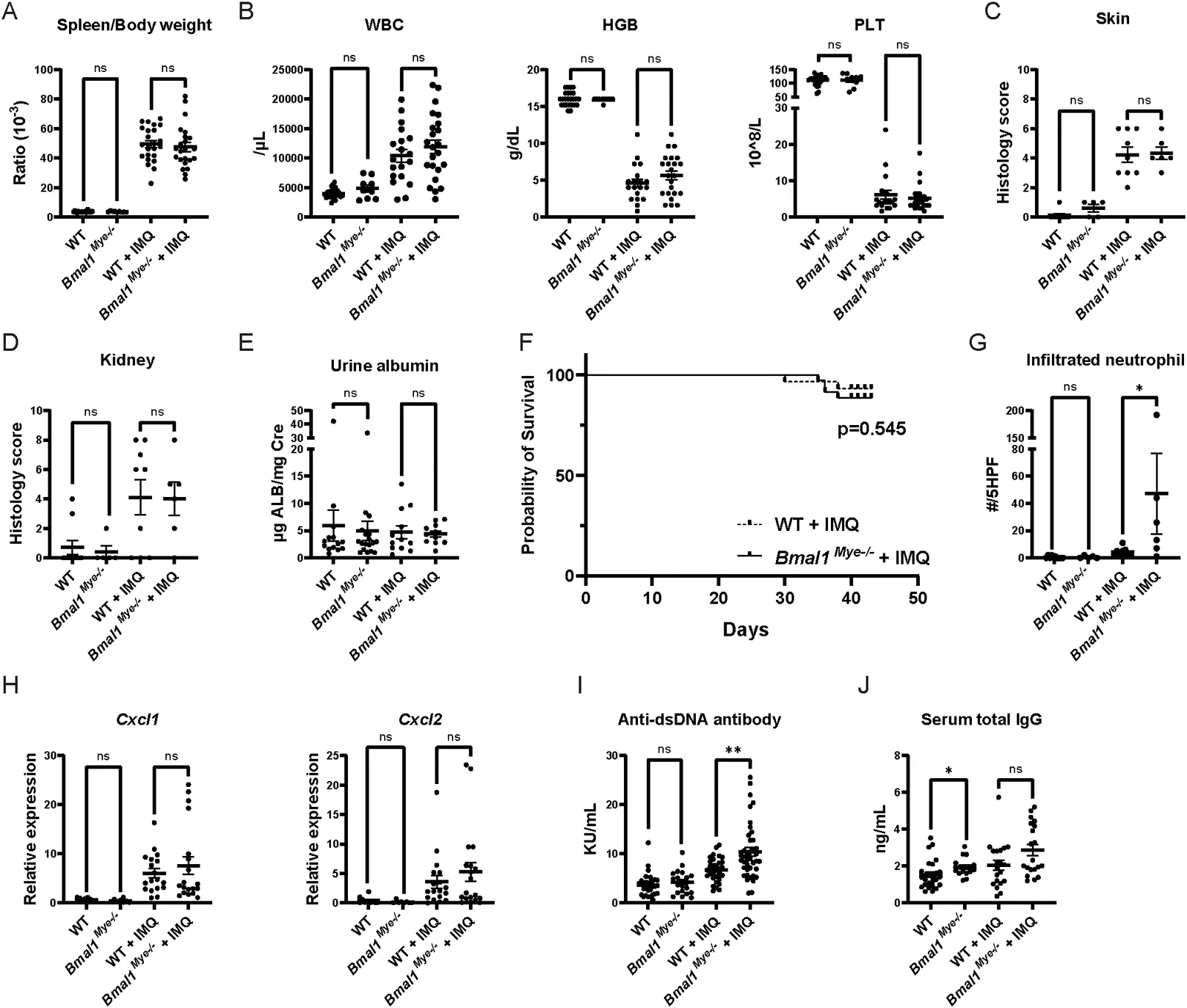
Lupus phenotype triggered by imiquimod in *Bmal1^Mye−/−^* and WT mice. **(A)** Spleen weight corrected by body weight in untreated and imiquimod (IMQ)-treated wild type (WT) and *Bmal1^Mye−/−^* mice. WT; n=25, *Bmal1^Mye−/−^*; n=9, WT + IMQ; n=24, *Bmal1^Mye−/−^* + IMQ; n=23. **(B)** Complete blood count (CBC) of peripheral blood. WT; n=25, *Bmal1^Mye−/−^*; n=9, WT + IMQ; n=24, *Bmal1^Mye−/−^* + IMQ; n=23. **(C, D)** Histology inflammation score in skin and kidney, respectively. WT; n=10, *Bmal1^Mye−/−^*; n=5, WT + IMQ; n=9, *Bmal1^Mye−/−^* + IMQ; n=6. **(E)** Urine albumin/creatinine ratio. WT; n=14, *Bmal1^Mye−/−^*; n=18, WT + IMQ; n=12, *Bmal1^Mye−/−^* + IMQ; n=11. **(F)** Survival rates analyzed by the Kaplan–Meier curve. WT + IMQ; n=29, *Bmal1^Mye−/−^* + IMQ; n=35. **(G)** Number of neutrophils per five high-power fields (HPF) of H&E staining in lesional skin. WT; n=10, *Bmal1^Mye−/−^*; n=5, WT + IMQ; n=9, *Bmal1^Mye−/−^* + IMQ; n=6. **(H)** Chemokine expression levels in skin (ear) lesion. Expression levels of the C–X–C motif chemokine ligand (Cxcl) 1 and Cxcl2 were assessed by qPCR. WT; n=10, *Bmal1^Mye−/−^*; n=8, WT + IMQ; n=18, *Bmal1^Mye−/−^* + IMQ; n=19. **(I, J)** Serum concentration of anti-ds DNA and total IgG, respectively. WT; n=25, *Bmal1^Mye−/−^*; n=16, WT + IMQ; n=20, *Bmal1^Mye−/−^* + IMQ; n=20. Bars in the graphs represent mean + SEM. The statistical analysis was done using unpaired t-test for spleen weight, CBC, urine albumin, and qPCR and Mann–Whitney test for histology score, infiltrated neutrophil count, anti-ds DNA antibody, total IgG. Log-rank test was used for survival rate analysis. *p<0.05, **p<0.01, ns, not significant. WBC, white blood cell; HGB, hemoglobin; PLT, platelet.

After 6 weeks of epicutaneous IMQ cream, WT and *Bmal1^Mye−/−^* mice displayed similar induction of splenomegaly, leukocytosis, anemia, thrombocytopenia, dermatitis, and mild glomerulonephritis as previously described for this model (**Figures 1A–D**). As in other studies using mice in C57BL6-background (10, 11), significant proteinuria was not observed (**Figure 1E**) and mortality rates were similar between groups during the treatment (**Figure 1F**).

**Figure 2:**
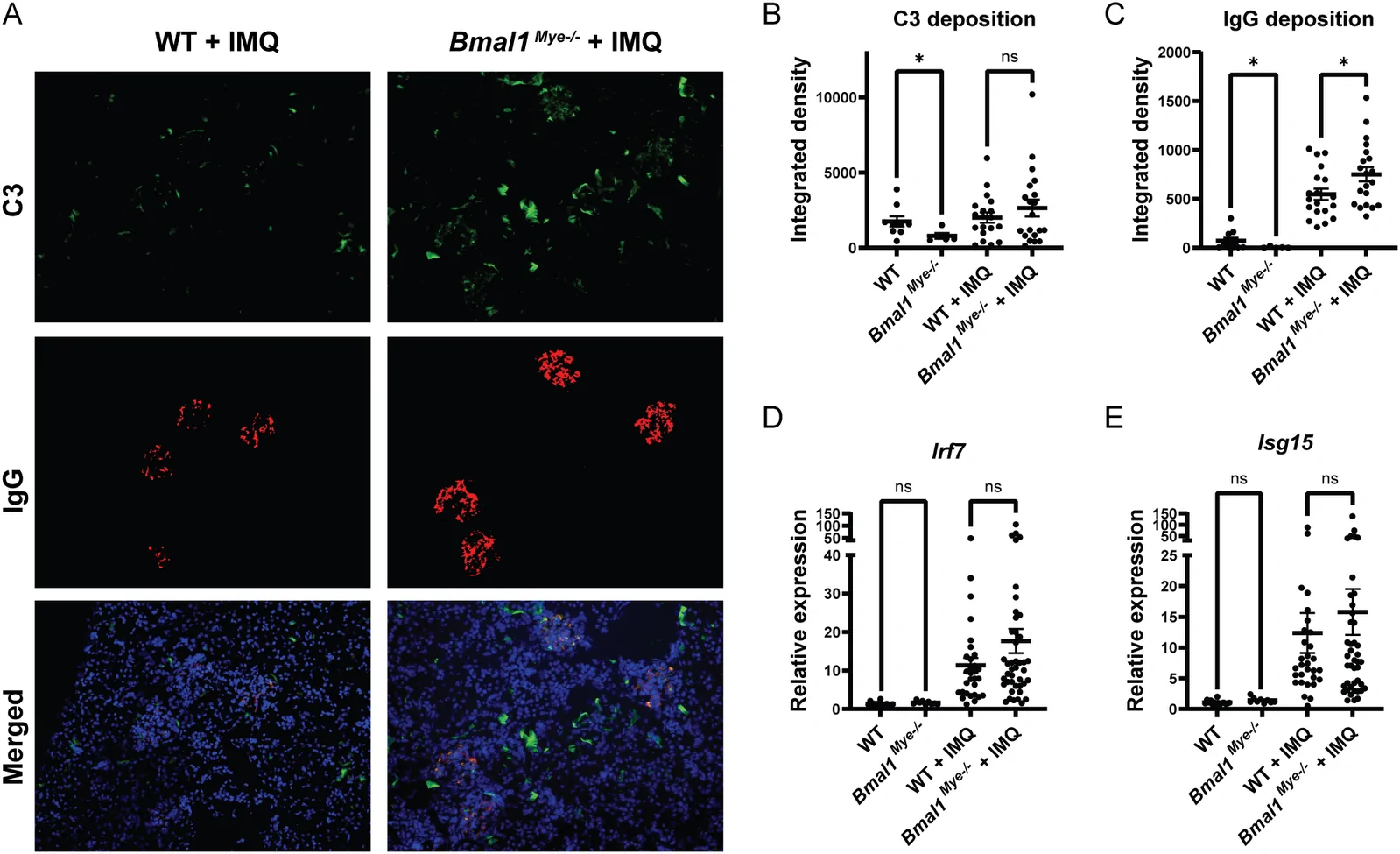
Immune complex deposition and inflammatory response in kidneys are modulated by Bmal1. **(A)** Representative images of kidney immunofluorescent staining of immune complex deposition. C3 (green), IgG (red), and Hoechst (blue). **(B, C)** Fluorescent intensity of C3 and IgG, respectively, quantified by ImageJ software. WT; n=9, *Bmal1^Mye−/−^*; n=5, WT + IMQ; n=19, *Bmal1^Mye−/−^* + IMQ; n=20. **(D, E)** Expression levels of interferon regulatory factor (Irf)7 **(D)** and interferon-stimulated gene (Isg)15 **(E)** were assessed by qPCR. WT; n=15, *Bmal1^Mye−/−^*; n=9, WT + IMQ; n=30, *Bmal1^Mye−/−^* + IMQ; n=43. Bar graphs represent mean + SEM. The statistical analysis was done using unpaired t-test for qPCR results, and Mann–Whitney test for immune complex deposition. *p<0.05, ns, not significant.

When assessing histology of the IMQ-treated local skin lesions, there was increased neutrophilic infiltration in the *Bmal1^Mye−/−^* mice compared with WT (**Figure 1G**), whereas there were similar degrees of skin inflammation (**Figure 1C**) and tissue chemokine levels (**Figure 1H**). This supports that Bmal1 regulates migratory capacity of myeloid cells in inflammatory milieus (3). Furthermore, IMQ-treated *Bmal1^Mye−/−^* mice displayed significantly higher levels of serum anti-dsDNA (**Figure 1I**) whereas serum total IgG (**Figure 1J**) and other autoantibodies (anti-RNP and anti-SS-A/Ro, **Supplementary Figure 1**) were not significantly different compared with IMQ-treated WT. Moreover, increased renal immune complex deposition was observed in IMQ-treated *Bmal1^Mye−/−^* compared with WT (**Figures 2A–C**). In contrast, expression levels of tissue type I interferon-stimulated genes (ISGs) did not significantly differ between both groups of mice (**Figures 2D, E**).

Next, we performed flow cytometry analysis of splenocytes to assess if Bmal1 regulates immune cell subsets. While no significant differences were found between WT and *Bmal1^Mye−/−^* in percentages of neutrophils, monocytes, conventional dendritic cells (cDCs), plasmacytoid DCs (pDCs), and CD4^+^ and CD8^+^ T cells (**Supplementary Figures 2A–F**), significant increases in the percentage of CD19^+^ B cells was observed in IMQ-treated *Bmal1^Mye−/−^* mice when compared with IMQ-treated WT mice (**Supplementary Figure 2G**), alongside a non-significant trend for increased splenic CD19^−^CD138^+^ plasma cells in *Bmal1^Mye−/−^* (**Supplementary Figure 2H**). There were no significant differences in follicular T helper cells (Tfh) and germinal center B cells (**Supplementary Figures 2I, J**). Furthermore, there were no differences in percentages of naïve CD19^+^ B cells, whereas marginal zone B cells were significantly lower in *Bmal1^Mye−/−^* (**Supplementary Figures 2K, L**). Since percentages of each cell fraction may not always reflect the actual expansion in cell numbers, we checked the total number of splenocytes, and found that it did not show any differences between WT and *Bmal1^Mye−/−^* mice (**Supplementary Figure 3**).

In summary, although the absence of Bmal1 in myeloid cells did not dramatically modify murine lupus symptoms during the 6 weeks of treatment, it led to increase anti-dsDNA antibody levels, renal immune complex deposition, and enhanced dermal tissue neutrophil infiltration.

### 3.2 Autoantigen generation is not altered in IMQ-treated *Bmal1^Mye−/−^*

**Figure 3:**
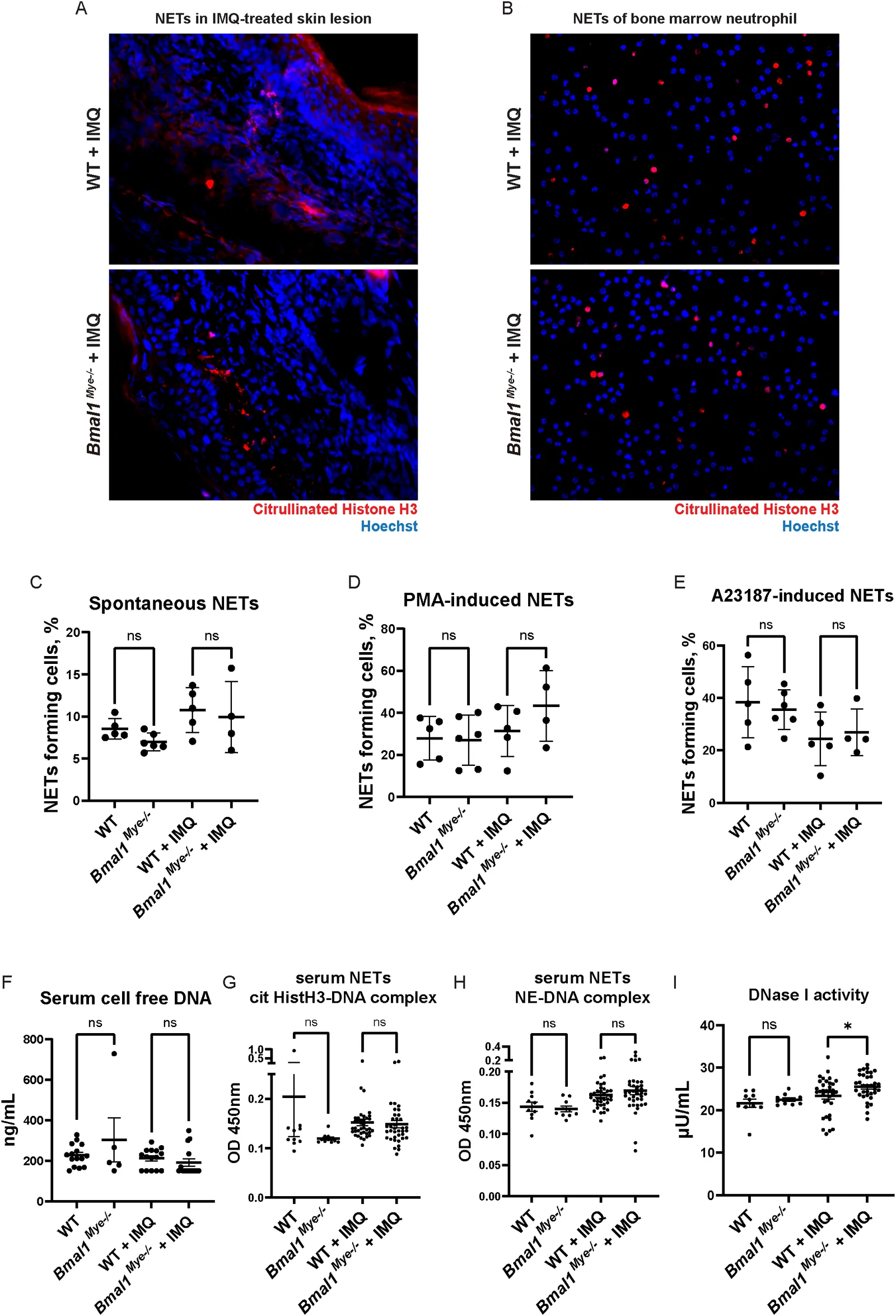
NET forming activity and DNA antigen levels are not regulated by Bmal1. **(A)** NETs in IMQ-treated local skin lesion. Frozen sections of imiquimod-treated ears were stained with anti-citrullinated histone H3 antibody (red) and Hoechst (blue). **(B)** Immunofluorescent staining of spontaneous NET formation of bone marrow neutrophils. Neutrophils were incubated on cover slips in serum-free RPMI media for 3h. NETs were stained with anti-citrullinated histone H3 antibody (red), and intra- and extracellular DNA was counterstained by Hoechst (blue). **(C–E)** Quantification of NETs with semi-automated live cell imaging method. Bone marrow neutrophils were incubated in serum-free RPMI media with or without stimulation. The percentage of cells externalizing NETs was calculated at 5 hours (C; spontaneous NETs, D; PMA-induced NETs) and 3 hours (E; A23187-induced NETs). WT; n=5, *Bmal1^Mye−/−^*; n=6, WT + IMQ; n=5, *Bmal1^Mye−/−^* + IMQ; n=4. **(F)** Cell-free DNA in mouse serum. WT; n=15, *Bmal1^Mye−/−^*; n=5, WT + IMQ; n=15, *Bmal1^Mye−/−^* + IMQ; n=15. **(G, H)** Serum NET complexes. cit HistH3-DNA complex **(G)** and NE-DNA complex **(H)** were quantified by sandwich ELISA. WT; n=10, *Bmal1^Mye−/−^*; n=10, WT + IMQ; n=38, *Bmal1^Mye−/−^* + IMQ; n=37. **(I)** Serum DNase I activity. WT; n=10, *Bmal1^Mye−/−^*; n=10, WT + IMQ; n=34, *Bmal1^Mye−/−^* + IMQ; n=34. Bars in the graphs represent mean + SEM. The statistical analysis was done using Mann–Whitney test. ns, not significant; WT, wild type; IMQ, imiquimod; NETs, neutrophil extracellular traps; PMA, phorbol-12-myristate-13-acetate; cit HistH3, citrullinated histone H3; NE, neutrophil elastase. *p<0.05.

We considered that increased autoantigen generation by neutrophils in *Bmal1^Mye−/−^* would contribute to increased autoantibody and immune complex formation. As higher numbers of neutrophils infiltrate, the IMQ-treated local skin lesions of *Bmal1^Mye−/−^* and this gene are known to modulate neutrophil biology (26), we assessed NET formation in the IMQ-treated local skin lesions. However, no significant differences in skin lesion NETs were detected by immunofluorescence (**Figure 3A**). While bone marrow neutrophils from IMQ-treated mice have enhanced spontaneous NET generation, no difference was observed between WT and *Bmal1^Mye−/−^* (**Figures 3B, C**). Responsiveness to PMA or to the A23187 calcium ionophore, both of which are inducers of NET formation, was not affected, although a non-statistically significant trend was seen for PMA-induced NETs (**Figures 3D, E**). Consistent with these findings, serum cell-free DNA and serum NET remnant levels, which can be used as surrogate markers of cell death and NET formation *in vivo*, were also similar between both groups of mice (**Figures 3F–H**). Since decreased serum DNase I activity has been reported and may contribute to increase autoantigen burden in human SLE patients (27), we tested serum DNase I activity in the mice. There was a statistically significant increase in DNase I activity in IMQ-treated *Bmal1^Mye−/−^* (**Figure 3I**), which may reduce autoantigen half-life in *Bmal1^Mye−/−^*. These results do not support that enhanced autoantibody and immune complex generation in *Bmal1^Mye−/−^* are the result of increased autoantigen generation.

### 3.3 Bone marrow neutrophil maturation is impaired in IMQ-treated *Bmal1^Mye−/−^* mice, in association with enhanced April production

**Figure 4:**
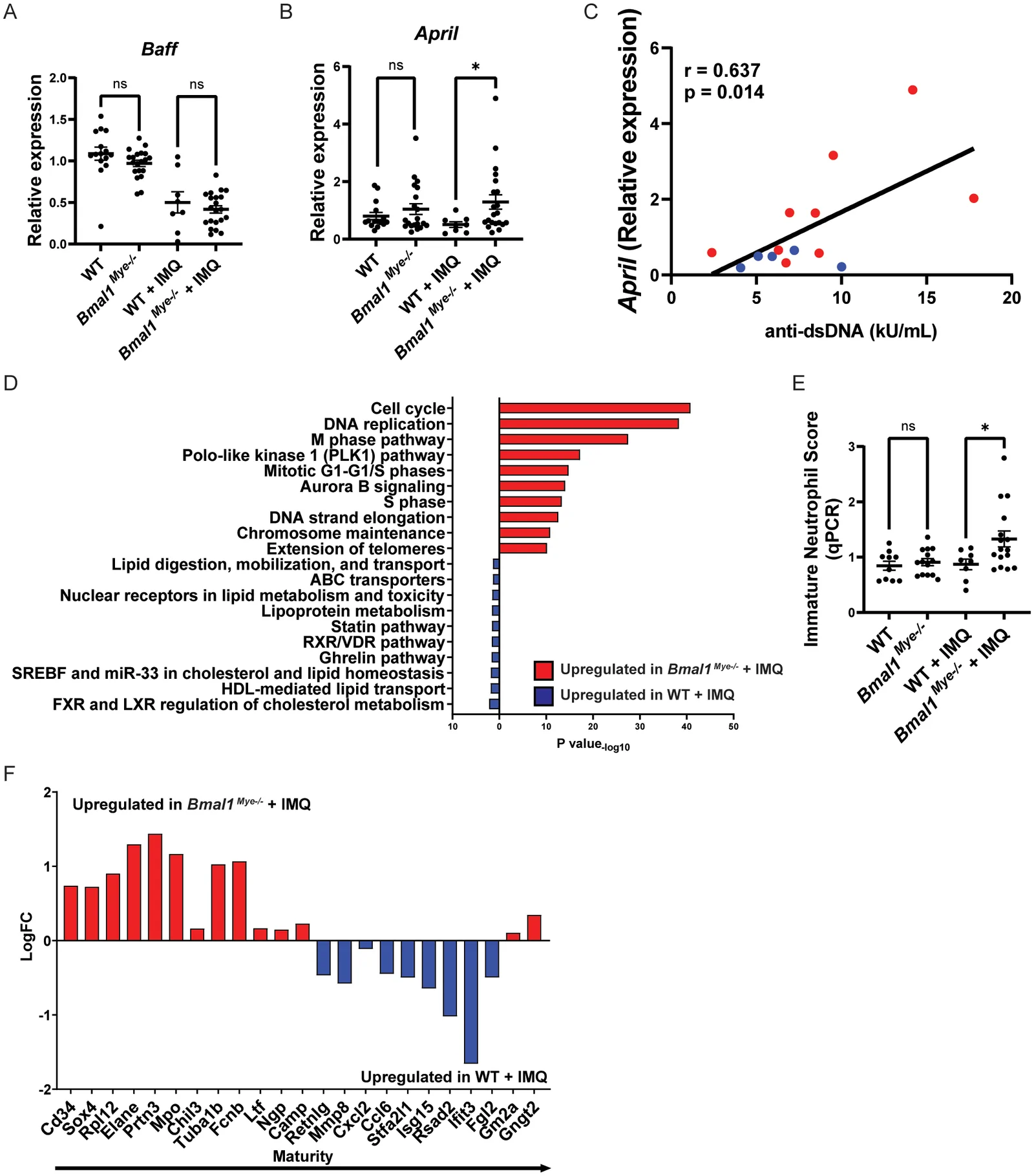
*April* expression and maturation status of neutrophils and their modulation by Bmal1. **(A, B)** mRNA expression of *Baff* and *April* in bone marrow neutrophils, evaluated by qPCR. WT; n=15, *Bmal1^Mye−/−^*; n=20, WT + IMQ; n=8, *Bmal1^Mye−/−^* + IMQ; n=21. **(C)** Correlation between *April* mRNA expression in bone marrow neutrophils and serum anti-dsDNA levels in IMQ-treated mice. WT + IMQ (blue dot); n=5, *Bmal1^Mye−/−^* + IMQ (red dot); n=9. **(D)** Pathway enrichment analysis of IMQ-treated WT and *Bmal1^Mye−/−^*. **(E)** Immature neutrophil score in bone marrow neutrophils calculated based on relative expression of 7 genes (*Mpo*, *Elane*, *Bpi*, *Ctsg*, *Prtn3*, *Camp*, and *Defa4*) analyzed by qPCR. WT; n=10, *Bmal1^Mye−/−^*; n=14, WT + IMQ; n=8, *Bmal1^Mye−/−^* + IMQ; n=16. **(F)** Expression pattern of genes that are representative of neutrophil maturation status. Genes associated with immaturity are on the left and those associated with mature status are on the right. Calculation was done with RPKM of each gene. Bars in the graphs represent mean ± SEM. The statistical analysis was done using unpaired t-test for qPCR and immature neutrophil score, and Pearson correlation coefficient for correlation analysis. *p<0.05, ns, not significant.

**Figure 5:**
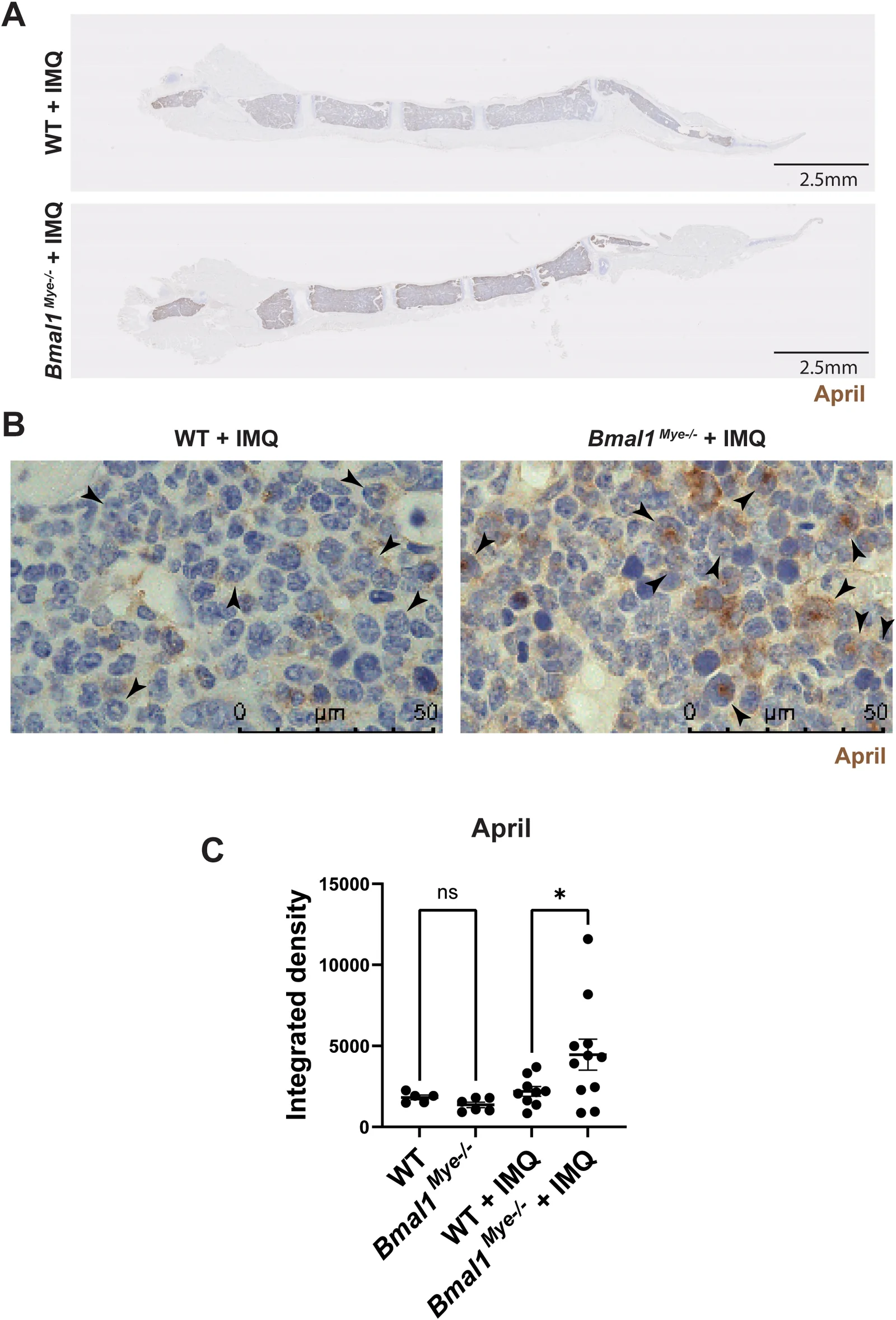
Immunohistochemistry staining for the expression of April in mouse bone marrow. **(A)** Representative lower magnification (0.82×) images of IMQ-treated WT and *Bmal1^Mye−/−^* sternum bone marrow. April was stained with anti-mouse April antibody. **(B)** Higher-magnification (100×) images. April expressed in immature neutrophils (arrowhead) is increased in IMQ-treated *Bmal1^Mye−/−^*. **(C)** April levels in whole bone marrow were quantified with ImageJ and compared. WT; n=5, *Bmal1^Mye−/−^*; n=5, WT + IMQ; n=9, *Bmal1^Mye−/−^* + IMQ; n=11. The statistical analysis was done using Mann–Whitney test. *p<0.05, ns, not significant.

As it has been reported that lupus bone marrow neutrophils can affect B-cell development through enhanced type I IFN, BAFF, and APRIL synthesis (2), we evaluated the expression of mRNAs encoding for these molecules in mouse bone marrow neutrophils. We found that *April* expression was significantly higher in IMQ-treated *Bmal1^Mye−/−^* neutrophils whereas there were no differences in *Baff* expression (**Figures 4A, B**). There was a non-significant trend for increased *Ifna4* in *Bmal1^Mye−/−^*, independent of IMQ treatment (**Supplementary Figure 4**). The neutrophil *April* expression level in the bone marrow was significantly associated with serum anti-dsDNA antibody levels in IMQ-treated mice (**Figure 4C**). Bulk RNA sequencing (RNA-seq) analysis of bone marrow neutrophils showed that the number of differentially expressed genes was small when the mice were not treated (**Supplementary Figure 5A**) but was enhanced when the mice were treated with IMQ (**Supplementary Figure 5B**). Pathway enrichment analysis revealed that genes associated with mitosis were upregulated in bone marrow neutrophils from IMQ-treated *Bmal1^Mye−/−^* (**Figure 4D**). The expression level of genes associated with neutrophil differentiation/maturation status (19–21) suggested higher levels of immature neutrophils in the IMQ-treated *Bmal1^Mye−/−^* mice (**Figures 4E, F**; **Supplementary Figure 6**). These observations are consistent with previous findings that reported the expression of *APRIL* being higher in immature myeloid cells (28). To confirm findings at the protein level, we attempted to quantify serum April levels, but they were under the detection level (data not shown). Bone marrow immunohistochemistry (IHC) showed that more April was detected in *Bmal1^Mye−/−^* bone marrow following IMQ treatment when compared with WT (**Figure 5A**). This increased April expression in immature neutrophils was observed at the protein level (**Figures 5B, C**). In contrast, splenic mRNA expression levels of *Baff* and *April* were similar between IMQ-treated WT and *Bmal1^Mye−/−^* (**Supplementary Figure 7**). The maturation status of bone marrow neutrophils in the absence of IMQ did not differ between WT and *Bmal1^Mye−/−^* (**Supplementary Figure 8**). Furthermore, the expression levels of *April* in bone marrow monocytes from IMQ-treated WT and *Bmal1^Mye−/−^* were comparable (**Supplementary Figure 9**).

We then incubated splenic plasma cells with supernatants from neutrophil cultures obtained from bone marrows from IMQ-treated mice. There was a non-statistically significant trend for IMQ-treated *Bmal1^Mye−/−^* neutrophil supernatants to increase the plasma cell survivals when compared with IMQ-treated WT neutrophil supernatants. A blocking anti-April monoclonal antibody reduced the survival of plasma cells in both IMQ-treated WT and KO mice (**Supplementary Figure 10**).

Taken together, these observations suggest that Bmal1 regulates neutrophil maturation in the bone marrow in murine models of lupus. Immature neutrophils increase in IMQ-treated *Bmal1^Mye−/−^* mice and produce more April. As such, it is possible that Bmal1 negatively regulates plasma cell dysregulation, at least in part, by controlling neutrophil differentiation and maturation and April synthesis in inflammatory conditions.

### 3.4 *BMAL1* expression level in human lupus peripheral neutrophils associates with clinical parameters

**Figure 6:**
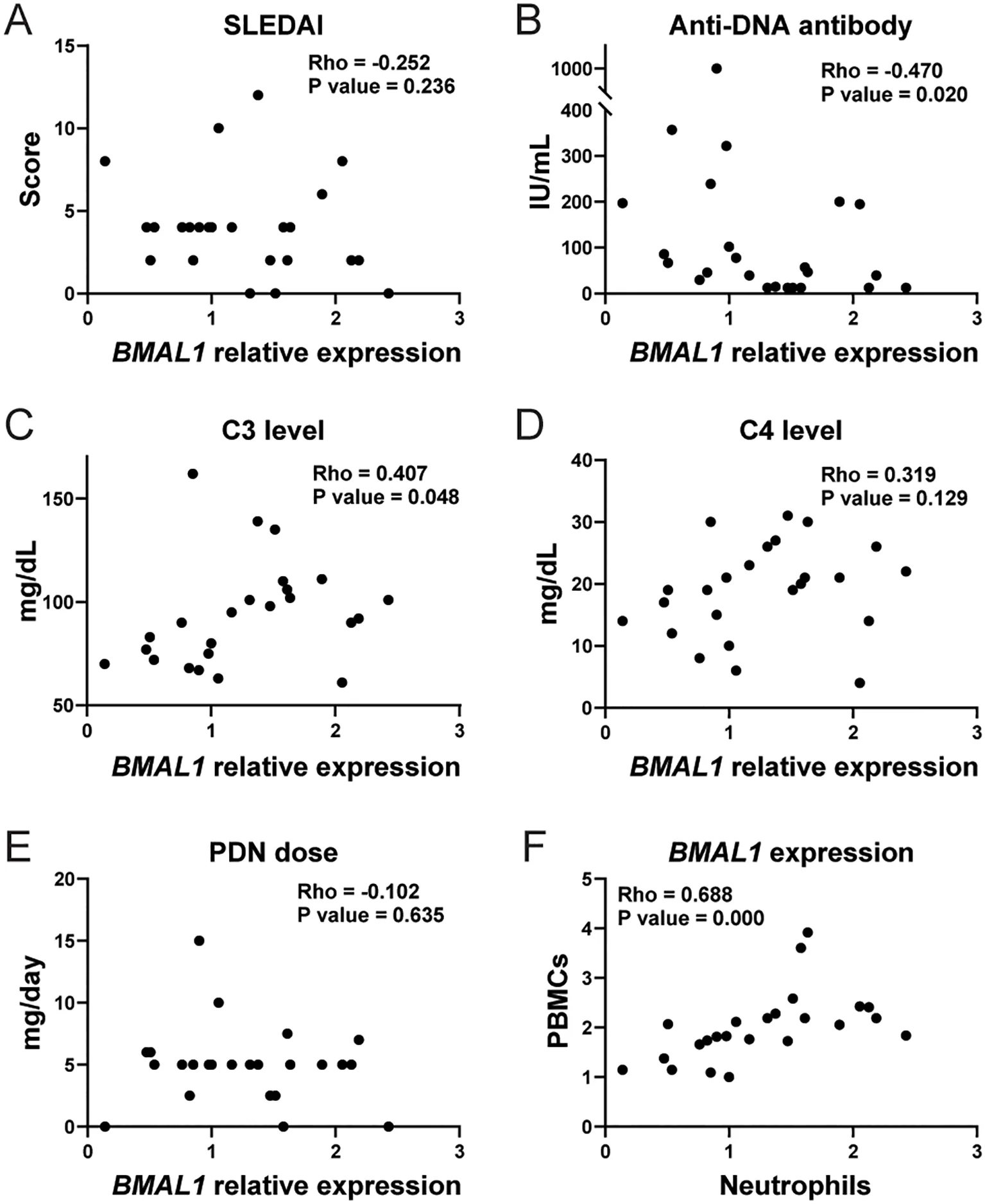
Correlation between clinical parameters and BMAL1 expression level in human SLE peripheral blood neutrophils. Correlation between *BMAL1* mRNA expression in peripheral blood neutrophils from human SLE patients (n= 24) and SLEDAI **(A)**, serum anti-ds DNA level **(B)**, serum C3 level **(C)**, serum C4 level **(D)**, and daily dose of oral prednisone (PDN) **(E)**. **(F)** Correlation between *BMAL1* expression in peripheral blood mononuclear cells (PBMCs) and neutrophils. The statistical analysis was done using Spearman’s rank correlation coefficient for correlation analyses.

We assessed if lupus clinical parameters correlate with *BMAL1* expression in peripheral blood neutrophils obtained from SLE patients. The patients’ demographics and clinical characteristics are shown in **Supplementary Table 2**. SLE neutrophils showed variability in *BMAL1* expression levels, which negatively associated with serum anti-dsDNA levels and positively with C3 levels, whereas SLEDAI and C4 showed no association (**Figures 6A–D**). When *BMAL1* expression levels were compared between patients that were negative or positive for other autoantibodies, they were significantly lower in anti-Sm-positive patients and tended to be lower in anti-RNP-positive patients, whereas there was no association with anti-SS-A/Ro- and anti-SS-B/La-positive patients (**Supplementary Figure 11**). We next assessed the association of *BMAL1* expression with corticosteroid dose, but no correlation was detected (**Figure 6E**; mean daily prednisone dose in this cohort was 4.96 ± 3.1 mg/day, mean + SEM). *BMAL1* expression levels correlated with neutrophils and PBMCs (**Figure 6F**), suggesting that the circadian clock is well-synchronized in peripheral white blood cells. Plasma levels of APRIL protein and expression levels of *APRIL* mRNA did not correlate with *BMAL1* expression levels in human neutrophils (**Supplementary Figures 12A, B**). This may be because peripheral blood neutrophils are mostly mature. Furthermore, plasma APRIL levels did not correlate with anti-dsDNA levels (**Supplementary Figure 12C**). When SLE and healthy donor APRIL levels were compared, they were higher in SLE, but the difference was not statistically significant (**Supplementary Figure 12D**). Overall, the data in lupus patients is consistent with findings in murine lupus suggesting that BMAL1 may negatively regulate autoantibody production.

## 4 Discussion

We report that *Bmal1*, a clock gene, negatively regulates autoantibody production in a TLR-7/8-dependent model of lupus. Lack of *Bmal1* in this model was associated with impaired neutrophil maturation and higher April synthesis in bone marrow. Furthermore, expression of *BMAL1* in human SLE peripheral blood neutrophils negatively associated with autoantibody levels, which is consistent with the mouse experiments.

It has been well-established that the immune system is affected by circadian rhythms. In autoimmune diseases, for example, morning stiffness is a well-known manifestation of rheumatoid arthritis. Optimizing the timing of drug administration according to target oscillation patterns, or clock genes themselves, may represent promising treatment targets in chronic inflammatory conditions (29). However, it remains largely unknown how dysregulation of clock genes affects human autoimmune disease.

*Bmal1* expression in neutrophils is high in the resting phase, which is daytime in nocturnal animals (including mice) and nighttime in diurnal animals (including humans) (3, 30). Adrover et al. showed that the aging of peripheral blood neutrophils is induced by Cxcr2 and antagonized by Cxcr4 signaling. *Bmal1* promotes cell aging by increasing the expression of Cxcl2, which stimulates Cxcr2 in an autocrine manner. As a result, in mice, fresh and active neutrophils are in circulation during the nighttime (active phase), and aged neutrophils are removed from circulation during the daytime (resting phase). Neutrophils defective of *Bmal1* are kept fresh and display enhanced infiltration of tissues (3). Given the evidence implicating neutrophils in the pathogenesis of lupus, we hypothesized that disruptions in neutrophils’ circadian rhythm through *Bmal1* disruption may be pathogenic in SLE.

A previous report showed that *Bmal1* germline KO mice displayed worsening of IMQ-induced psoriasis and systemic type I IFN responses (31). However, we did not see significant differences in IMQ-treated local skin lesion and systemic IFN responses in the lupus model induced by IMQ. These differences could be explained by differences in duration and localization of topical IMQ treatment as well as germline versus conditional KO systems. In our study, the most striking differences were observed in levels of serum anti-dsDNA and renal immune complex deposition. At first, we hypothesized that increased anti-dsDNA antibody levels were related to enhanced autoantigen generation through NET forming activity because it has been reported that neutrophils defective in Bmal1 form more NETs (26). However, we did not see any difference in NET formation both *in vitro* and *in vivo* in this lupus animal model. This discrepancy may be attributable to differences in NET-inducing and NET quantification methods and, potentially, in the animal model used. We also quantified serum cell-free DNA and DNase I activity, and the results of these analyses did not suggest increased autoantigen generation or persistence in the absence of Bmal1. It is possible that other sources of DNA may be important for this model, including apoptotic cell-derived microparticles, and this could be explored in future studies (32). We found that *April* expression in bone marrow neutrophils was significantly higher in IMQ-treated *Bmal1^Mye−/−^* than in IMQ-treated WT. APRIL is a cytokine produced by various cells including immature myeloid cells in both mouse and human systems (28). APRIL binds to the B-cell maturation antigen (BCMA) and the transmembrane activator and CAML-interactor (TACI) and is engaged in long-lived plasma cell survival in bone marrow (33). Previous studies in lupus mouse models have suggested that plasma cells in both spleen and bone marrow produce anti-dsDNA (34). Considering that there were no differences in germinal center reaction and in Baff/April production in the spleen of IMQ-treated mice, and that human plasma APRIL levels did not associate with anti-dsDNA serum levels, it is possible that increased neutrophil-derived APRIL is utilized primarily in the bone marrow and that differences in anti-dsDNA levels are secondary to bone marrow B-cell lineage changes. Indeed, previous studies have shown that SLE bone marrow neutrophils distinctly synthesize B-cell factors such as APRIL and BAFF, modulating B-cell ontogeny (2). Previous reports also showed that April deficiency does not reduce total IgG levels in mice (35, 36). In agreement with these findings, total IgG elevation was not observed in *Bmal1^Mye−/−^* compared with WT mice. It is possible that APRIL production by bone marrow neutrophils may act more specifically on autoreactive plasma cells in the bone marrow. Future studies should explore this possibility.

IMQ-treated *Bmal1^Mye−/−^* neutrophils displayed a more immature phenotype compared with WT. These observations are consistent with a previous report that showed that APRIL is produced by immature myeloid cells (28). Of note, the difference in maturation was seen only when mice were treated with IMQ. This suggests that Bmal1 regulation of immune responses may be more relevant in the context of inflammatory conditions and innate immune activation. Given that the genes regulating steady-state and emergency granulopoiesis are different (37), granulopoiesis in lupus may be controlled differently than in the steady state. In SLE, BMAL1 may be involved only in granulopoiesis in inflammatory conditions such as emergency granulopoiesis.

On the other hand, *Ifna4* was increased in *Bmal1^Mye−/−^*, irrespective of IMQ treatment, even though there was no statistically significant difference. There was also no difference in *Baff* expression level between WT and *Bmal1^Mye−/−^*. These results differ from a previous report that showed increased expressions of IFNα, BAFF, and APRIL in human SLE bone marrow neutrophils (2). Further research is warranted to better understand the complexities of this regulation.

Analysis of human samples was overall consistent with the murine lupus data. It was recently reported that shortened sleep duration increases SLE risk (7). Sleep deprivation results in reduced *BMAL1* expression in human peripheral leukocytes (38). Although we do not have available data on the sleeping status of the patients in our cohort and did not sequentially test APRIL expression, our data suggest that reduction of *BMAL1* expression, potentially caused by sleep deprivation or other factors that disrupt circadian rhythm (pain, steroid use, etc.), may have pathogenic consequences. However, we do not have available longitudinal data of *BMAL1* expression and, given that these can be affected by sleep cycle and other factors (38), the levels of this molecule may fluctuate during the disease course. As high autoantibody levels are associated with high cumulative lupus disease activity (39, 40), one could propose that reducing autoantibody levels by modulating circadian rhythms may be beneficial for disease control. Therefore, BMAL1 and circadian rhythm regulation could become promising therapeutic targets.

Our study has several limitations. We have not demonstrated a direct causal relationship between April synthesis by neutrophils and changes in B-cell numbers and autoantibody production. Although the plasma cell survival assay showed small differences between WT and *Bmal1^Mye−/−^*, it was not statistically significant, and it is unclear whether it represents *in vivo* situation. It is possible that our findings are model-dependent. Preferably, confirmatory studies using genetically prone lupus models, such as MRL/*lpr* or NZB/W F1, should be performed in the future. Given that the KO mice used are myeloid conditional KO mice, it is possible that other myeloid cells beyond neutrophils are involved in the dysregulation of immune responses. Previous reports showed that Bmal1 affects the number of monocytes/macrophages infiltrating atherosclerotic lesions (41, 42). As such, it is possible that other types of myeloid cells could be implicated in our model. It is unclear whether the correlation between anti-ds DNA antibody levels and bone marrow neutrophil April expression is the main factor explaining the differences in B cell biology between WT or *Bmal1^Mye−/−^*. Indeed, the difference in DNase I activity in *Bmal1^Mye−/−^* may reflect effects on other myeloid cells, given that DNase1L3, one of DNase1 family proteins, can be secreted by them (43). As such, increased serum anti-dsDNA levels in *Bmal1^Mye−/−^* may not be attributed solely to neutrophils. However, since there were no differences in *April* expression in bone marrow monocytes, we consider that it is more likely that April released from bone marrow neutrophils modulates autoantibody production in this model. On the other hand, the relevance of the increased percentage of CD19^+^ B cell in *Bmal1^Mye−/−^* spleen is unclear. No specific fraction was increased in our evaluation, and there was no statistically significant difference in CD138^+^CD19^−^ plasma cells in spleen. Given that April does not act on mature naïve B cells (33), the expansion of B cells cannot be explained by April production in the bone marrow neutrophils. As such, further analysis is necessary to clarify these observations. Finally, the role of the higher number of *Bmal1^Mye−/−^* neutrophils that infiltrated inflammatory skin lesions remains unclear. Since a recent report showed that neutrophils are “primed” in UV-irradiated skin and then migrate into kidney (44), skin-infiltrated neutrophils may transmigrate into tissues and be involved in immune responses. Future studies should address the pathways involved in circadian rhythm dysregulation and aberrant autoimmune responses to answer these questions and observations.

In conclusion, the results of this study suggest that dysregulation in clock genes contributes to autoantibody production in lupus by abrogating neutrophil differentiation and increasing APRIL production by bone marrow neutrophils. These results have potential implications in regulating circadian rhythm to modulate inflammation in SLE and other autoimmune conditions.

## Data Availability

The datasets presented in this study can be found in online repositories. The names of the repository/repositories and accession number(s) can be found below: https://www.ncbi.nlm.nih.gov/geo/query/acc.cgi?acc=GSE279310, GSE279310.
