# Supplementary material for: The circadian clock gene BMAL1 modulates autoimmunity features in lupus
Source: Front Immunol. 2024 Nov 27;15:1465185. doi: 10.3389/fimmu.2024.1465185 (PMC11631884; doi:10.3389/fimmu.2024.1465185)
Supplement: Supplementary file 1 [file DataSheet1.docx]

Supplementary Figures


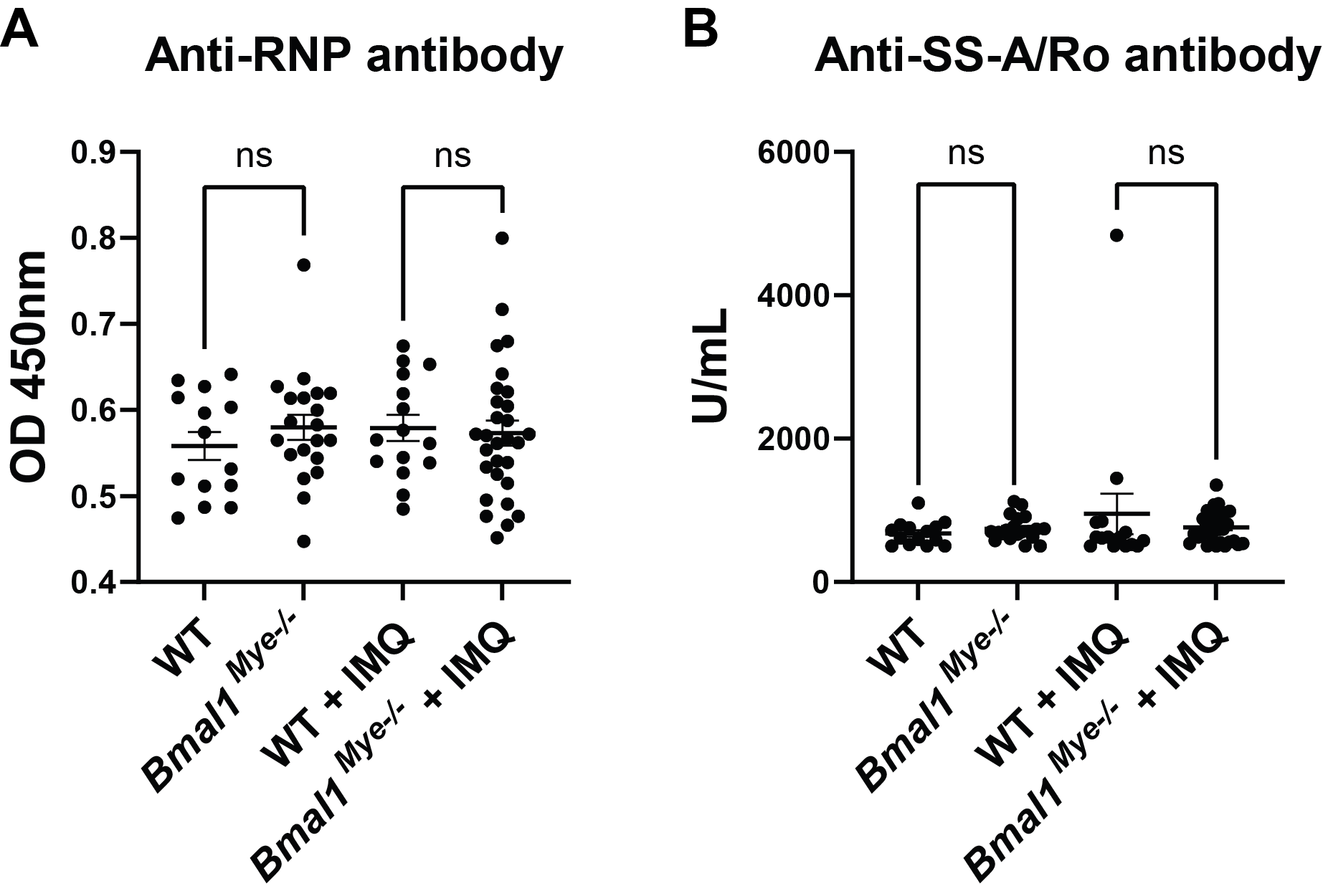


**Supplementary Figure 1.** **Serum anti-RNP and anti-SS-A/Ro antibody levels.**

Mouse serum anti-RNP antibody (A) and anti-SS-A/Ro antibody (B) levels were evaluated by ELISA. WT; n=14, *Bmal1^Mye−/−^*; n=20, WT + IMQ; n=15, *Bmal1^Mye−/−^* + IMQ; n=29. Bars represent mean + SEM. The statistical analysis was done using Mann-Whitney test. ns; not significant, OD; optical density, WT; wild type, IMQ; imiquimod.


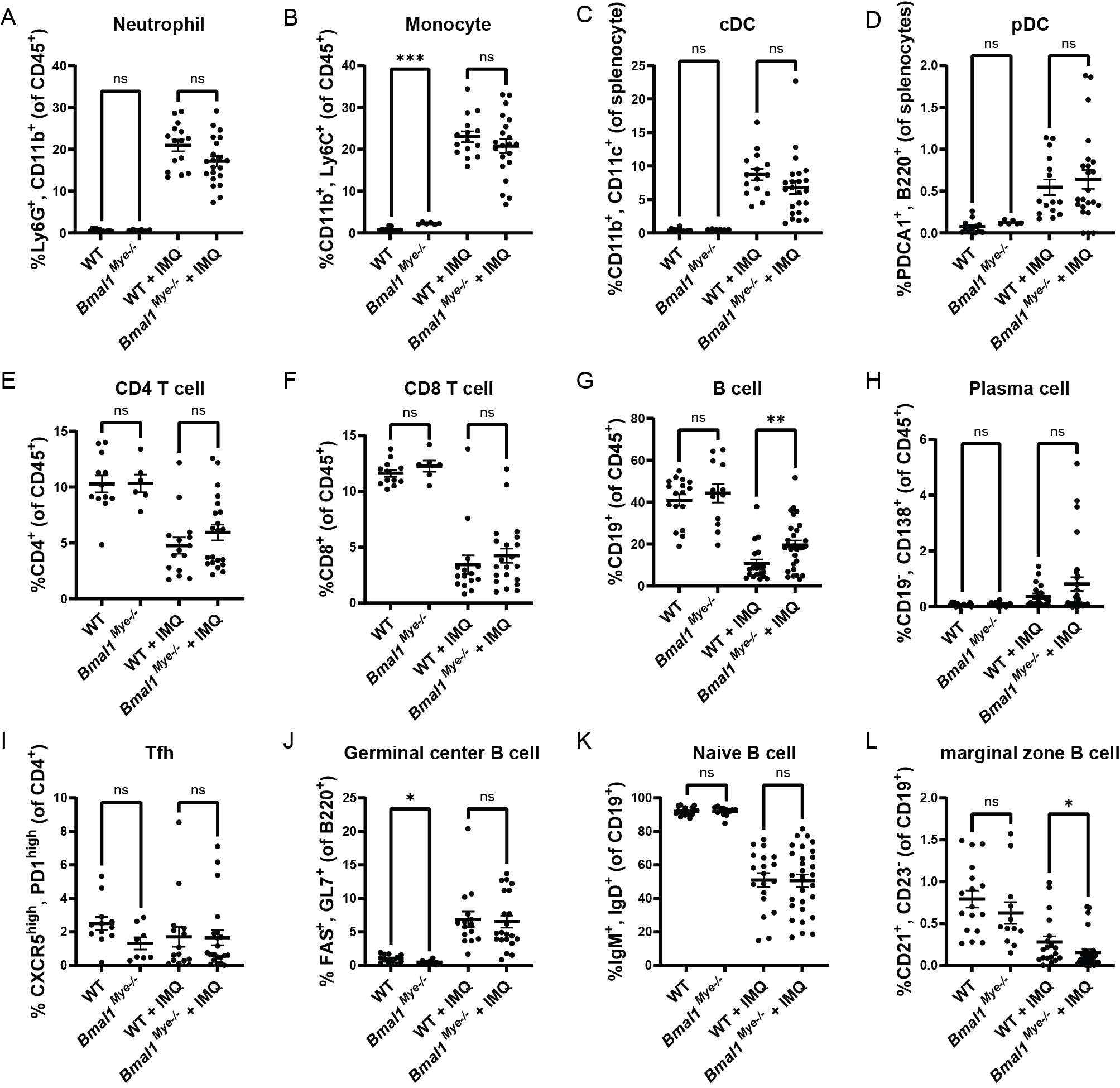


**Supplementary Figure 2.** **Flow cytometric analysis of splenocytes.**

Percentage of each cell fraction in spleen was evaluated by flow cytometry. Percentage of Ly6G^+^CD11b^+^ neutrophil (A), Ly6G^-^Ly6C^+^CD11b^+^monocyte (B), CD3^+^CD19^-^CD4^+^ T cell (E), CD3^+^CD19^-^CD8^+^ T cell (F), CD138^-^CD19^+^ B cell (G), and CD138^+^CD19^-^ plasma cell (H) in CD45 positive cells, CD19^-^CD11b^+^CD11c^+^ cDC (C) and CD11c^-^PDCA-1^+^B220^+^CD11c^+^ pDC (D) in live splenocytes, CXCR5^high^PD1^high^ Tfh in CD4 T cell (I), FAS^+^GL7^+^ germinal center B cell in B220^+^ B cell (J), and IgM^+^IgD^+^ naïve B cell (K) and CD21^+^CD23^-^ marginal zone B cell (L) in CD19^+^ B cell. (A-F) WT; n=12, *Bmal1^Mye−/−^*; n=6, WT + IMQ; n=15, *Bmal1^Mye−/−^* + IMQ; n=23. (G and H) WT; n=17, *Bmal1^Mye−/−^*; n=12, WT + IMQ; n=19, *Bmal1^Mye−/−^* + IMQ; n=29. (I and J) WT; n=12, *Bmal1^Mye−/−^*; n=8, WT + IMQ; n=15, *Bmal1^Mye−/−^* + IMQ; n=21. (K and L) WT; n=17, *Bmal1^Mye−/−^*; n=12, WT + IMQ; n=19, *Bmal1^Mye−/−^* + IMQ; n=29. Bars represent mean + SEM. The statistical analysis was done using Mann-Whitney test, *; p<0.05, ***; P<0.001, ns: not significant. cDC: conventional dendritic cells, pDC: plasmacytoid dendritic cells, Tfh; follicular T helper cell: WT; wild type, IMQ; imiquimod


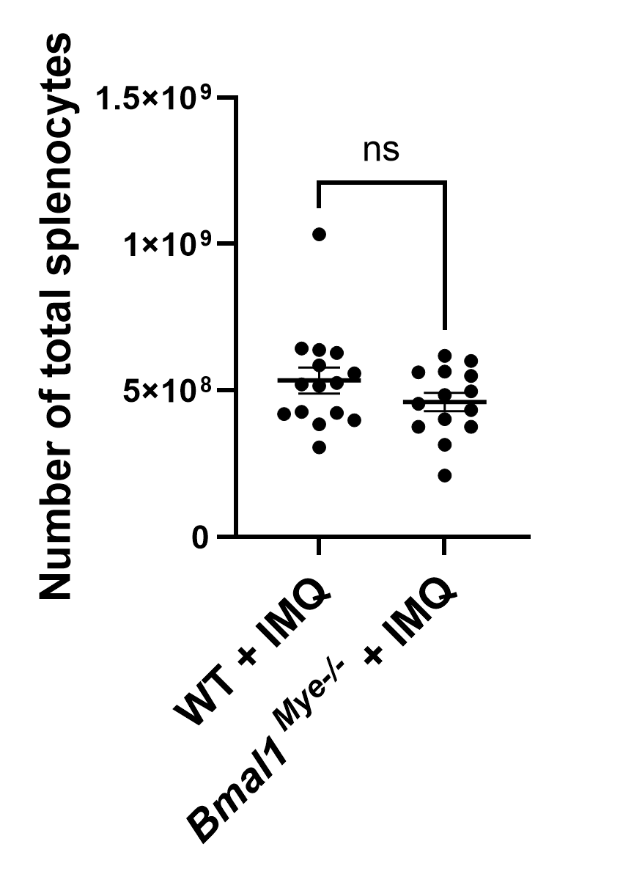


**Supplementary Figure 3.** **Absolute count of splenocytes by flow cytometry.**

WT + IMQ; n=15, *Bmal1^Mye−/−^* + IMQ; n=14. Bars represent mean + SEM. The statistical analysis was done using Mann-Whitney test, ns: not significant.


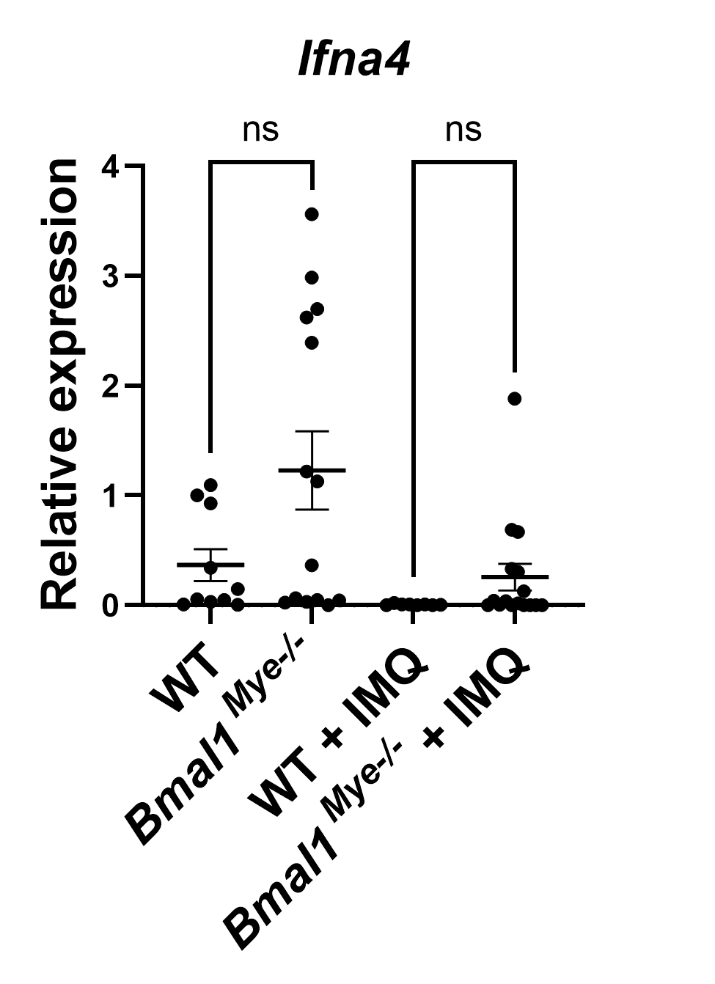


**Supplementary Figure 4.** **Interferon alpha-4 mRNA expression in bone marrow neutrophils.**

WT; n=10, *Bmal1^Mye−/−^*; n=14, WT + IMQ; n=8, *Bmal1^Mye−/−^* + IMQ; n=16. The statistical analysis was done using Mann-Whitney U test. ns; not significant


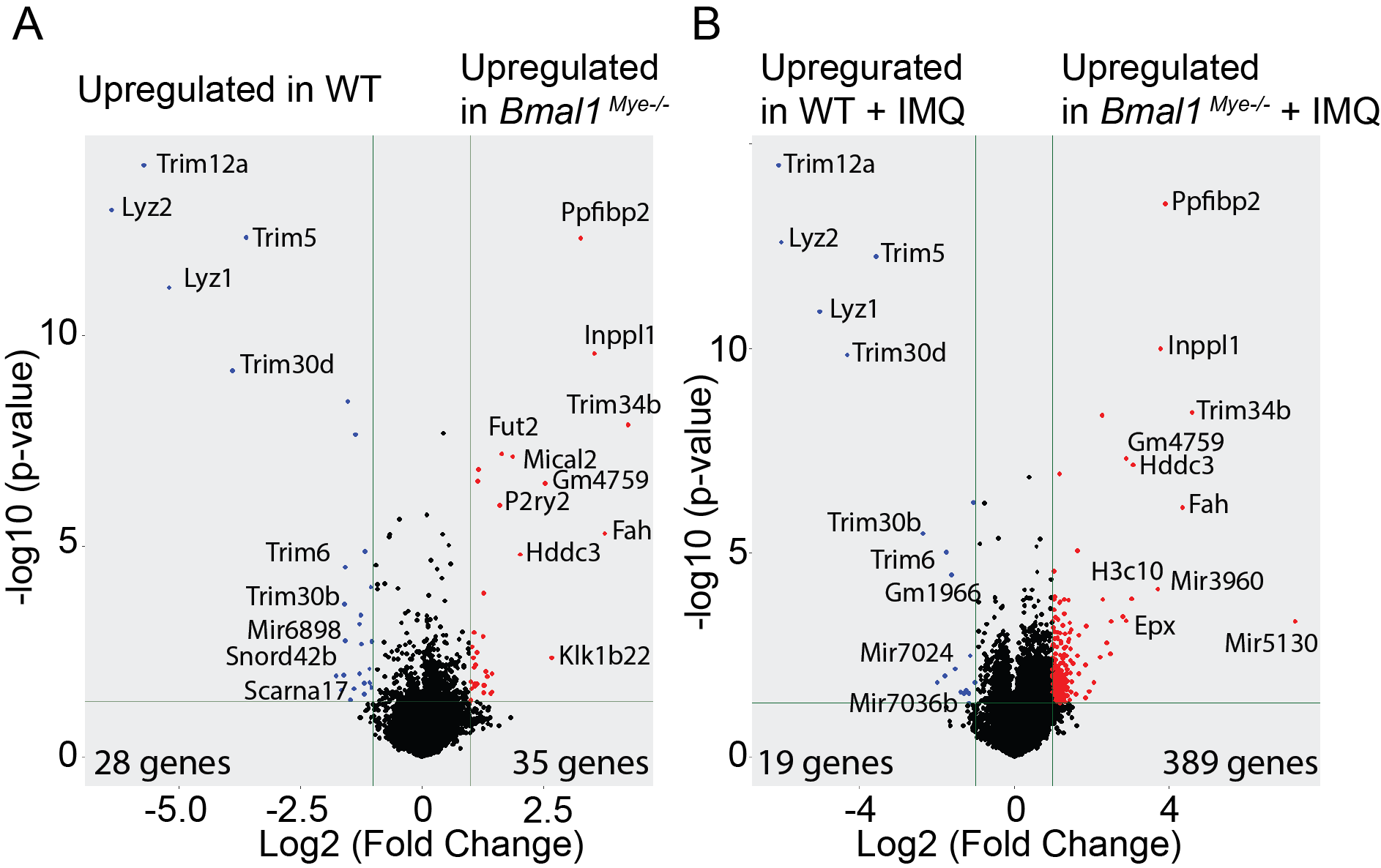


**Supplementary Figure 5**. **Volcano plot of differentially expressed genes in bone marrow neutrophils**

(A) Volcano plot showing differentially expressed genes (DEGs) obtained by bulk-RNA sequencing of bone marrow neutrophils comparing untreated WT and *Bmal1^Mye−/−^*. (B) Volcano plot of differentially regulated genes between IMQ-treated WT and *Bmal1^Mye−/−^*. WT; n=5, *Bmal1^Mye−/−^*; n=５, WT + IMQ; n=5, *Bmal1^Mye−/−^* + IMQ; n=5.


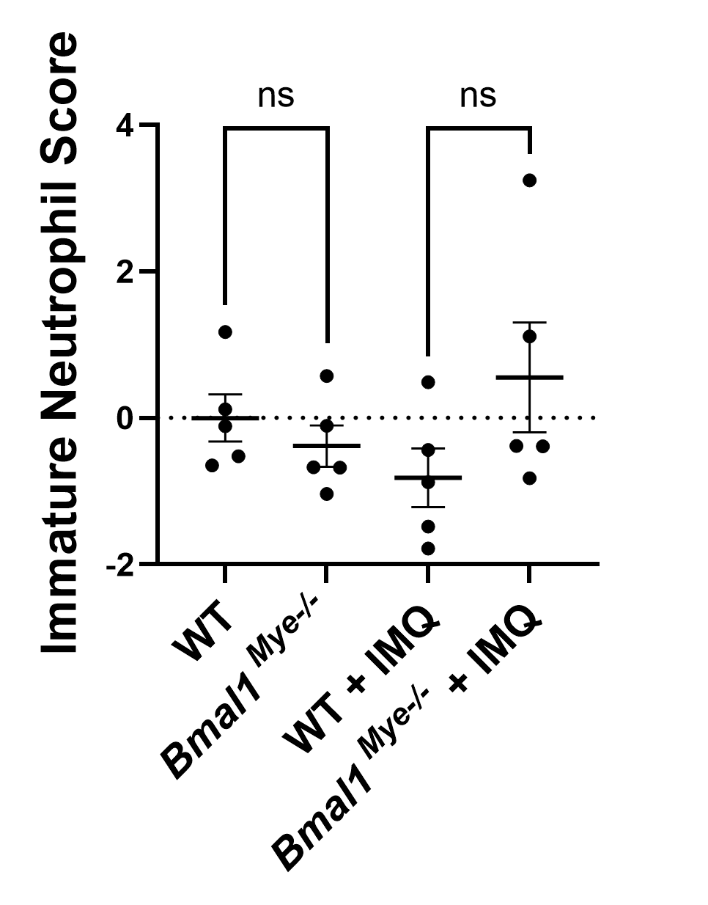


**Supplementary Figure 6**. **Immature neutrophil score of bone marrow neutrophils**

Score was calculated using RPKM of 7 genes (*Mpo*, *Elane*, *Bpi*, *Ctsg*, *Prtn3*, *Camp*, and *Defa4*) in bulk RNA-seq data. n=5 in each group. The statistical analysis was done using Mann-Whitney test. ns; not significant


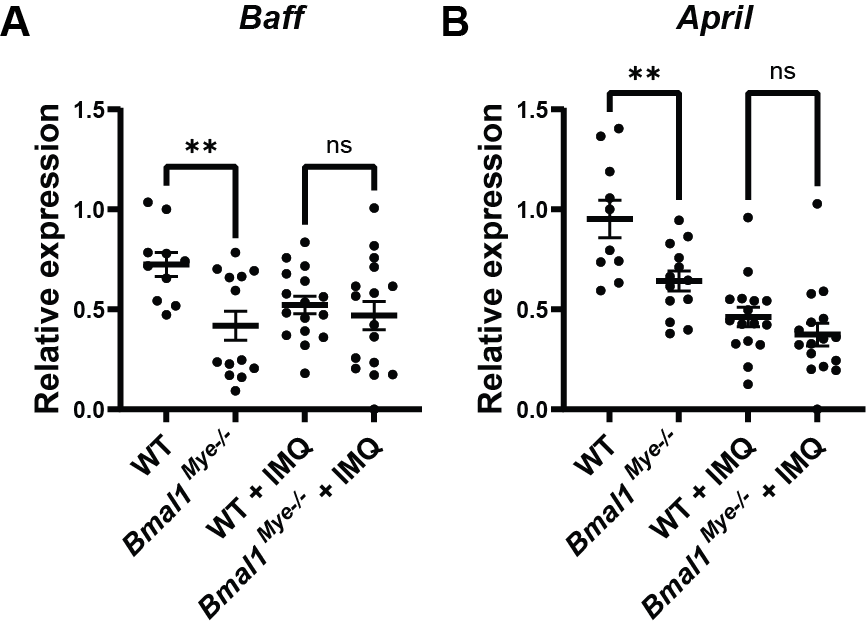


**Supplementary Figure 7**. **mRNA expression of** **B cell stimulating factors in spleen.** Expression level of *Baff* (A) and *April* (B) in spleen was evaluated by qPCR. WT; n=10, *Bmal1^Mye−/−^*; n=13, WT + IMQ; n=16, *Bmal1^Mye−/−^* + IMQ; n=16. Bars in the graphs represent mean + SEM. The statistical analysis was done using unpaired t-test. **; p<0.01 ns; not significant, WT; wild type, IMQ; imiquimod.


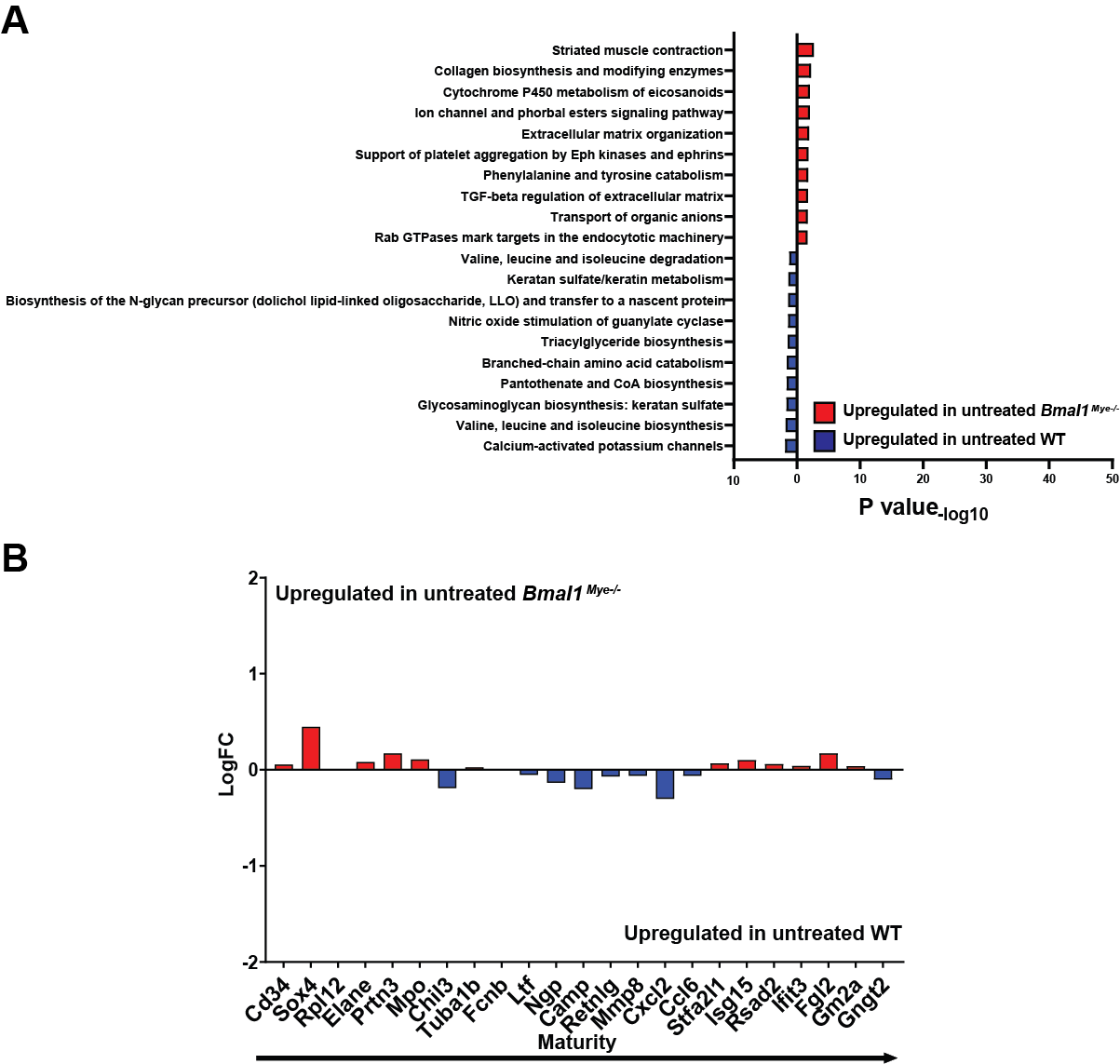


**Supplementary Figure 8**. **Comparison of gene expression of bone marrow neutrophils from untreated mice.**

(A) Pathway analysis of differentially expressed genes in bone marrow neutrophils from untreated wild type (WT) and *Bmal1^Mye−/−^* mice. Red and blue bars represent pathways enriched in *Bmal1^Mye−/−^* and WT, respectively. (B) Differential expression of genes that are representative of maturation status of neutrophil. Genes are sorted from left to right from less to more maturation status. WT; n=5, *Bmal1^Mye−/−^*; n=5. Red and blue bars represent genes upregulated in *Bmal1^Mye−/−^* and WT, respectively. WT; wild type


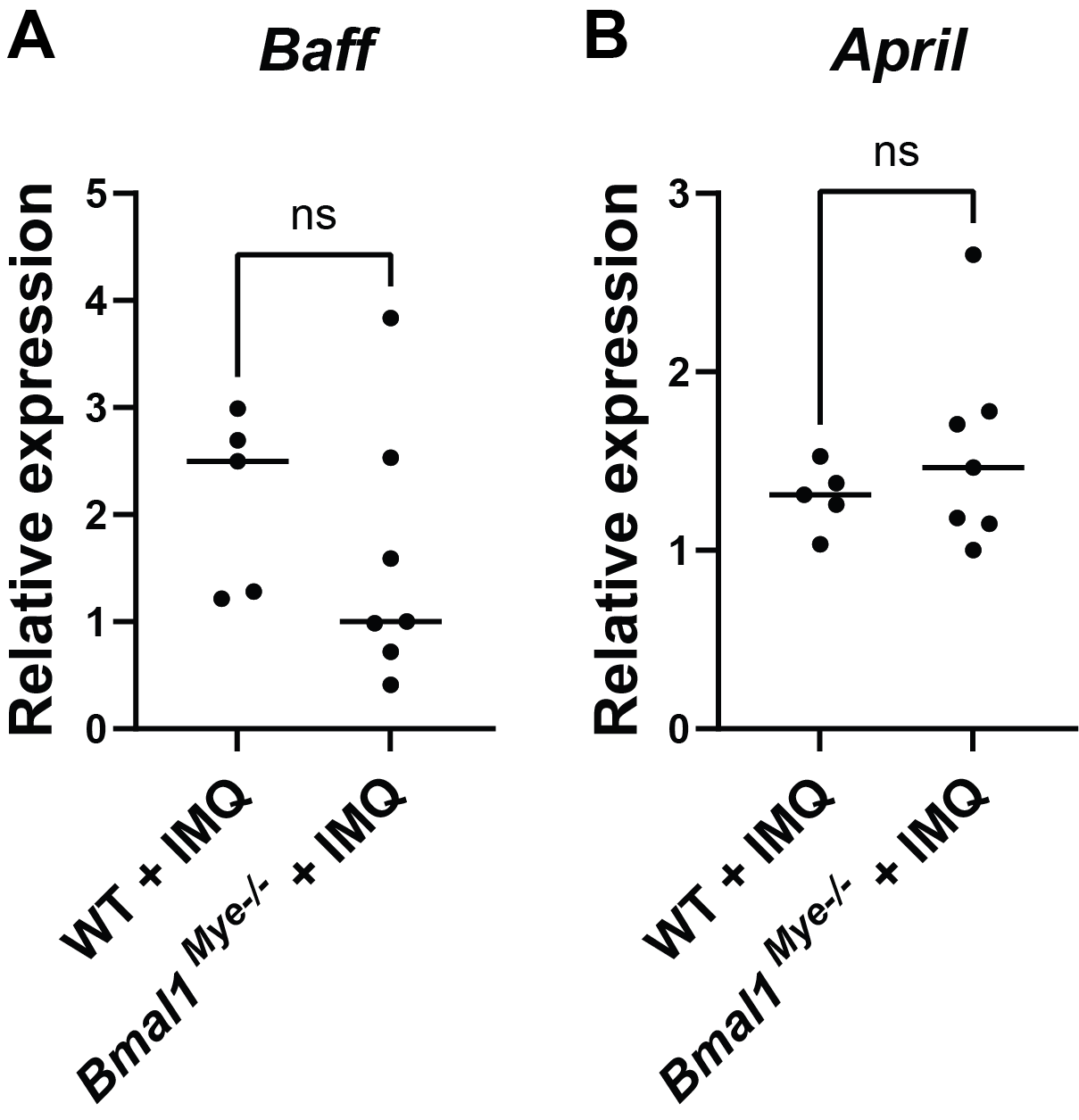


**Supplementary Figure 9**. **Expression levels of *Baff* and *April* in bone marrow monocytes.**

mRNA expression of *Baff* (A) and *April* (B) in mouse bone marrow monocytes was evaluated by qPCR. WT + IMQ; n=5, *Bmal1^Mye−/−^* + IMQ; n=7. Analysis was done using unpaired t-test. ns; not significant


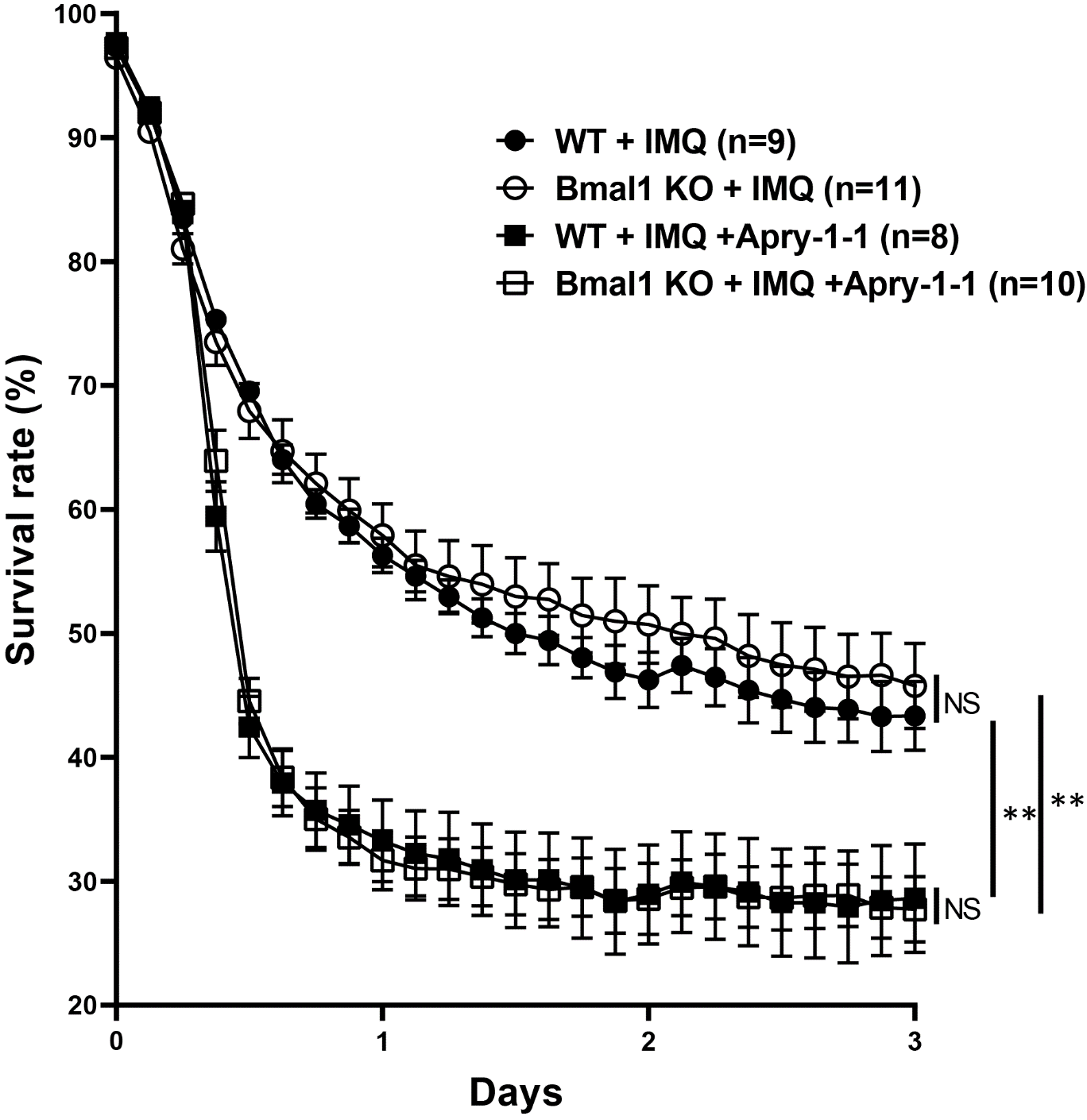


**Supplementary Figure 10. Survival rate of splenic plasma cells**

Splenic plasma cells were incubated in supernatant of bone marrow neutrophils from IMQ-treated WT and *Bmal1^Mye−/−^* in the presence or absence of April-neutralizing antibody (Apry-1-1). Bars in the graphs represent mean ± SEM. The statistical analysis was done using repeated measures two-way ANOVA with Bonferroni correction. **; p<0.01, NS; not significant


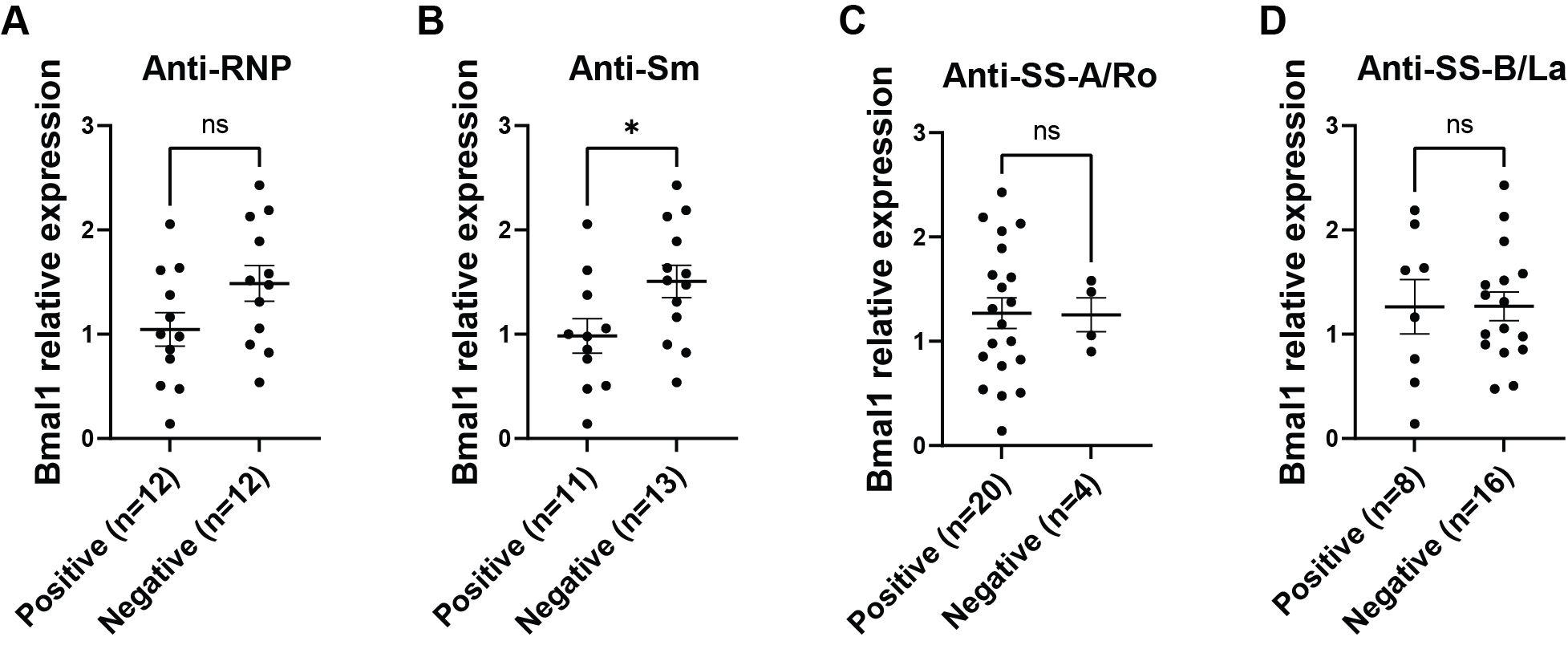


**Supplementary Figure 11**. **Association of positivity of autoantibodies and *BMAL1* expression in peripheral neutrophils from SLE patients.**

Relative expression level of *BMAL1* in peripheral neutrophils that was determined by qPCR was compared between positive and negative group of each autoantibody; anti-RNP antibody (A), anti-Sm antibody (B), anti-SS-A/Ro antibody (C), and anti-SS-B/La antibody (D). Bars in the graphs represent mean + SEM. The statistical analysis was done using Mann-Whitney test. *; p<0.05, ns; not significant.


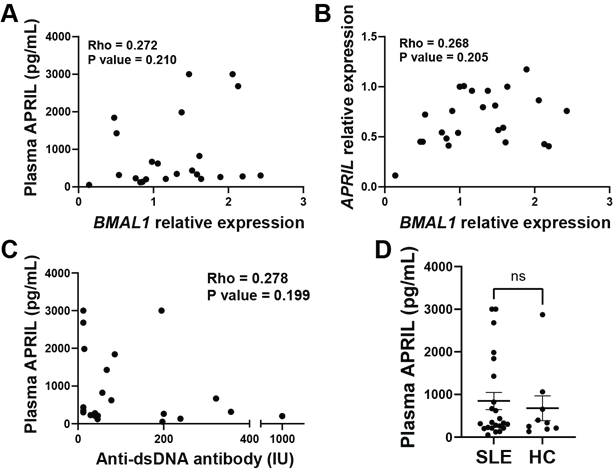


**Supplementary Figure 12**. **Expression and plasma levels of APRIL in human peripheral blood.**

Plasma APRIL cytokine levels were assessed by commercially available ELISA using plasma from SLE patients and healthy controls (HCs). (A and B) Comparison between *BMAL1* expression in peripheral neutrophils and plasma APRIL level (A) and APRIL expression in peripheral neutrophils (B). (C) Comparison between plasma APRIL levels and anti-dsDNA levels. (D) Plasma APRIL levels in SLE patients (n=24) and HCs (n=9). Bars in the graphs represent mean + SEM. The statistical analysis was done using Spearman's rank correlation coefficient for correlation analyses and Mann-Whitney test for the comparison between SLE and HC. ns; not significant.
