# Supplementary material for: The circadian clock gene BMAL1 modulates autoimmunity features in lupus
Source: Front Immunol. 2024 Nov 27;15:1465185. doi: 10.3389/fimmu.2024.1465185 (PMC11631884; doi:10.3389/fimmu.2024.1465185)
Supplement: Supplementary file 3 [file Table1.docx]

**Supplementary table 1. Histologic scoring of mouse kidneys**

| A | Initial evaluation |
| --- | --- |
|  | Slides evaluated for glomerular changes that are above that seen in normal aging mice that typically have expansion of mesangial matrix (MM) with aging. |
|  |  |
| B | Second evaluation: Semi-quantitative scoring |
|  | Glomeruli are graded for degree and distribution of hypercellularity (HC) and MM expansion and distribution and inflammation. Glomerulosclerosis is only graded for distribution. |
|  |  |
|  | Hypercellularity scores |
|  | 0: Evenly distributed nuclei in the mesangium and along capillary tufts |
|  | 1: Focal segmental expansion by clusters of cells |
|  | 2: Multifocal expansion by clusters of cells |
|  | 3: Global increase in cellularity |
|  |  |
|  | Mesangial matrix scores |
|  | 0: Glomerus typical of a young health mouse with the most prominent MM at the vascular pole and thinning out toward the urinary space |
|  | 1: Focal segmental expansion of matrix toward the urinary space |
|  | 2: Multifocal segmental expansions of matrix toward the vascular space |
|  | 3: Global expansion of matrix |
|  |  |
|  | Inflammation |
|  | 0: None |
|  | 1: Loosely packed neutrophilic infiltrates wit out tissue destruction |
|  | 2: Denser neutrophils with fibrin and necrosis |
|  | 3: Dense neutrophils which obscure normal architecture. |
|  |  |
|  | Distribution scores for all parameters were determined by an estimate of the number of glomeruli affected per kidney section |
|  | 0.      <1 % |
|  | 1.      1-25% |
|  | 2.      25-75% |
|  | 3.      >75% |
