# Supplementary material for: The circadian clock gene BMAL1 modulates autoimmunity features in lupus
Source: Front Immunol. 2024 Nov 27;15:1465185. doi: 10.3389/fimmu.2024.1465185 (PMC11631884; doi:10.3389/fimmu.2024.1465185)
Supplement: Supplementary file 4 [file Table2.docx]

**Supplementary table 2. Clinical characteristics of SLE patients included in this study**

| # | Sex | AGE | Ethnicity | *BMAL1* expression | SLEDAI | C3 (mg/dL) | C4 (mg/dL) | Anti-ds DNA (IU/mL) | RNP | Sm | SS-A/Ro | SS-B/La | CCP | PDN (mg/day) | Other drugs |
| --- | --- | --- | --- | --- | --- | --- | --- | --- | --- | --- | --- | --- | --- | --- | --- |
| 1 | F | 49 | Hispanic | 1.000 | 4 | 80 | 10 | 102 | + | + | + | - | ND | 5 | HCQ, MMF, BEL |
| 2 | F | 63 | Hispanic | 0.476 | 4 | 77 | 17 | 85.9 | + | + | + | - | ND | 6 | HCQ, AZA, |
| 3 | F | 40 | Hispanic | 0.540 | 4 | 72 | 12 | 357 | - | - | + | + | ND | 5 | HCQ, AZA, |
| 4 | F | 57 | Hispanic | 1.474 | 2 | 98 | 31 | <12.3 | - | - | - | - | ND | 2.5 | HCQ, TCZ, TAC |
| 5 | F | 40 | African American | 0.979 | 4 | 75 | 21 | 322 | + | + | + | - | ND | 5 | MMF |
| 6 | F | 36 | Hispanic | 0.901 | 4 | 67 | 15 | >1000 | - | - | - | - | ND | 15 | HCQ, MMF, BEL |
| 7 | F | 55 | Hispanic | 0.142 | 8 | 70 | 14 | 197 | + | + | + | + | ND | 0 | HCQ, AZA |
| 8 | F | 36 | Asian | 1.165 | 4 | 95 | 23 | 39.4 | + | - | + | + | ND | 5 | HCQ, MMF |
| 9 | F | 35 | Hispanic | 1.892 | 6 | 111 | 21 | 200 | - | - | + | - | ND | 5 | HCQ, MTX |
| 10 | F | 31 | African American | 1.376 | 12 | 139 | 27 | 14.7 | + | + | + | - | - | 5 | HCQ |
| 11 | F | 63 | Asian | 0.824 | 4 | 68 | 19 | 45.8 | - | - | + | - | - | 2.5 | AZA |
| 12 | F | 36 | Asian | 1.636 | 4 | 102 | 30 | 46.4 | + | - | + | + | ND | 5 | HCQ, MMF |
| 13 | F | 34 | Hispanic | 2.056 | 8 | 61 | 4 | 195 | + | + | + | + | - | 5 | HCQ, MTX |
| 14 | F | 43 | Hispanic | 1.613 | 2 | 106 | 21 | 56.9 | + | + | + | + | - | 7.5 | HCQ, BEL, CYC |
| 15 | F | 41 | African American | 2.189 | 2 | 92 | 26 | 39.5 | - | - | + | + | ND | 7 | HCQ, AZA |
| 16 | F | 55 | Caucasian | 0.853 | 2 | 162 | 30 | 239 | + | + | + | - | + | 5 | HCQ, LEF |
| 17 | F | 39 | Hispanic | 1.057 | 10 | 63 | 6 | 77.8 | - | + | - | - | ND | 10 | HCQ, MMF |
| 18 | M | 38 | Hispanic | 1.310 | 0 | 101 | 26 | <12.3 | - | - | + | - | ND | 5 | HCQ, AZA, MMF |
| 19 | F | 40 | Hispanic | 0.763 | 4 | 90 | 8 | 29.4 | + | + | + | + | ND | 5 | MMF |
| 20 | M | 59 | Caucasian | 2.428 | 0 | 101 | 22 | <12.3 | - | - | + | - | ND | 0 | HCQ |
| 21 | F | 42 | unknown | 1.580 | 4 | 110 | 20 | <12.3 | - | - | - | - | - | 0 | HCQ, MTX |
| 22 | F | 49 | Hispanic | 1.516 | 0 | 135 | 19 | <12.3 | - | - | + | - | ND | 2.5 | HCQ |
| 23 | F | 63 | Hispanic | 0.507 | 2 | 83 | 19 | 67 | + | + | + | - | ND | 6 | HCQ, AZA |
| 24 | F | 58 | Asian | 2.129 | 2 | 90 | 14 | <12.3 | - | - | + | - | ND | 5 | HCQ, AZA |

RNP; positivity of anti-RNP antibody, Sm; positivity of anti-Sm antibody, SS-A/Ro; positivity of anti-SS-A/Ro antibody, SS-B/La; positivity of anti-SS-B/La antibody, CCP: positivity of anti-CCP antibody, PDN; prednisone, ND; No data, HCQ; hydroxy chloroquine, MMF; mycophenolate mofetil, BEL: belimumab, AZA; azathioprine, TCZ; tocilizumab, TAC; tacrolimus, MTX; methotrexate, CYC; cyclophosphamide, LEF; leflunomide
